# Low‐Temperature Annealing Triggered Abnormal Strengthening in a Complex Concentration Alloy via Evolutive Short‐Range Ordering

**DOI:** 10.1002/advs.202506962

**Published:** 2025-07-03

**Authors:** Yihan Wang, Yuan Wu, Yong Yu, Yang He, Xinyang Yu, Xiongjun Liu, Hui Wang, Suihe Jiang, Xiaobin Zhang, Zhaoping Lu

**Affiliations:** ^1^ State Key Laboratory for Advanced Metals and Materials University of Science and Technology Beijing Beijing 100083 China; ^2^ Beijing Advanced Innovation Center for Materials Genome Engineering School of Materials Science and Engineering University of Science and Technology Beijing Beijing 100083 China

**Keywords:** complex concentration alloys, deformation mechanism, low‐temperature annealing, mechanical properties, medium entropy alloys, short‐range ordering

## Abstract

Low‐temperature annealing is traditionally employed to relieve residual stresses in metallic materials, typically resulting in softening. However, a novel finding in the face‐centered‐cubic (fcc) CoNiV medium entropy alloy (MEA) is reported, where low‐temperature annealing induces significant hardening without sacrificing ductility. Specifically, after annealing at 530 °C, the yield strength increases from 503 to 653 MPa, while maintaining plasticity of ≈60%. The comprehensive analysis reveals that this unexpected strengthening is attributed to the development of multi‐scale chemical short‐range orderings (SROs) during the annealing process. These SROs, particularly a newly formed L12‐type ordered structure (SRO‐2), enhance material strength by promoting dislocation slip planarity and reducing dislocation entanglement. This study demonstrates that low‐temperature annealing can effectively optimize atomic‐scale structures in complex alloys distinct from that in conventional alloys, thereby improving their mechanical properties. These findings extend the conventional understanding of annealing effects and highlight the potential for leveraging SROs to design high‐performance materials.

## Graphical abstract

Low‐temperature annealing enhances strength, ductility, and work hardening in CoNiV MEA. This originates from multi‐scale chemical short‐range orderings (SROs) formed during annealing, which promote dislocation slip planarity and reduce entanglement, enabling balanced mechanical properties through ordering strengthening.

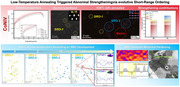

## 1 Introduction

The heat treatment of metallic materials, an ancient practice refined for thousands of years, plays an indispensable role in their process control, microstructure tailoring, and property optimization. Understanding the relationship between structure and property under the influence of temperature is of paramount importance. Traditional annealing processes, particularly those performed at medium to low temperatures below the recrystallization threshold, commonly known as stress‐relief annealing, are designed to alleviate residual stresses without forming any new phases. This process enhances the dimensional integrity of the material, thereby reducing the tendency for distortion and fracture.^[^ ^1^ ^]^ Nevertheless, this traditional methodology often leads to a certain degree of softening due to annihilation of dislocations. While effective for stress management, these inherent limitations of conventional thermal processing have spurred the need for innovative materials capable of overcoming softening while maintaining a balance between strength and ductility.

Recently, the development of complex concentration alloys (CCAs), such ashigh‐entropy alloys (HEAs) and MEAs, has garnered significant attention due to their potential to meet the growing demand for high‐performance materials.^[^ ^2^, ^3^ ^]^ Since their inception, CCAs have been regarded as a model of random solid solutions, where various types of constituent atoms are assumed to be randomly distributed at lattice sites due to the dominant effect of high configurational entropy over the enthalpy of compound formation.^[^ ^4^ ^]^ However, this assumption has recently been challenged due to oversimplified views about phase constitutions in CCAs,^[^ ^5^, ^6^, ^7^, ^8^ ^]^ highlighting the need for a more comprehensive understanding of the intricate relationship between properties and structures.

Atomic arrangements in CCAs are not at an ideal disordered state due to their large chemical complexity, including differences in atomic sizes, electronic structures, and mixing enthalpies.^[^ ^9^, ^10^, ^11^ ^]^ Instead, local atomic structures, particularly SROs were frequently observed as a result of complex interactions between constituent elements.^[^ ^12^, ^13^, ^14^, ^15^, ^16^ ^]^ Moreover, formation of SROs is thermodynamically and kinetically favorable in systems with high configuration entropy, which is considered as a common structural characteristic of CCAs.^[^ ^17^, ^18^ ^]^ Gradually, it is believed that tailoring SROs through appropriate alloying and composition adjustments can further optimize properties of CCAs.^[^ ^19^, ^20^ ^]^

In this study, we report a surprising finding: low‐temperature annealing in the fcc CoNiV MEA results in significant hardening without compromising plasticity. Specifically, yield strength increased by ≈30%, from 503 to 653 MPa, while maintaining ∼60% plasticity. By characterizing the intrinsic structural features and their evolution during annealing, we found that multi‐scale chemical SROs significantly developed during the low‐temperature annealing process in the CoNiV MEA. Our comprehensive characterization reveals that this hardening is attributed to the development of multi‐scale chemical SROs during annealing. These SROs not only enhance material strength but also promote dislocation slip planarity and reduce dislocation entanglement, maintaining large ductility and work‐hardening capability. Through profound understanding of the chemical complexity and structural metastability inherent in carefully selected CCA systems, we demonstrate that even a simple low‐temperature annealing process can remarkably optimize atomic‐scale structures. This finding reveals surprisingly advantageous approach for tailoring atomic‐scale structures in complex alloys, thereby establishing a novel and efficient strategy for enhancing mechanical properties in CCA systems.

## 2 Results and Discussion

### 2.1 Strengthening Induced by Low‐Temperature Annealing in CoNiV

**Figure 1:**
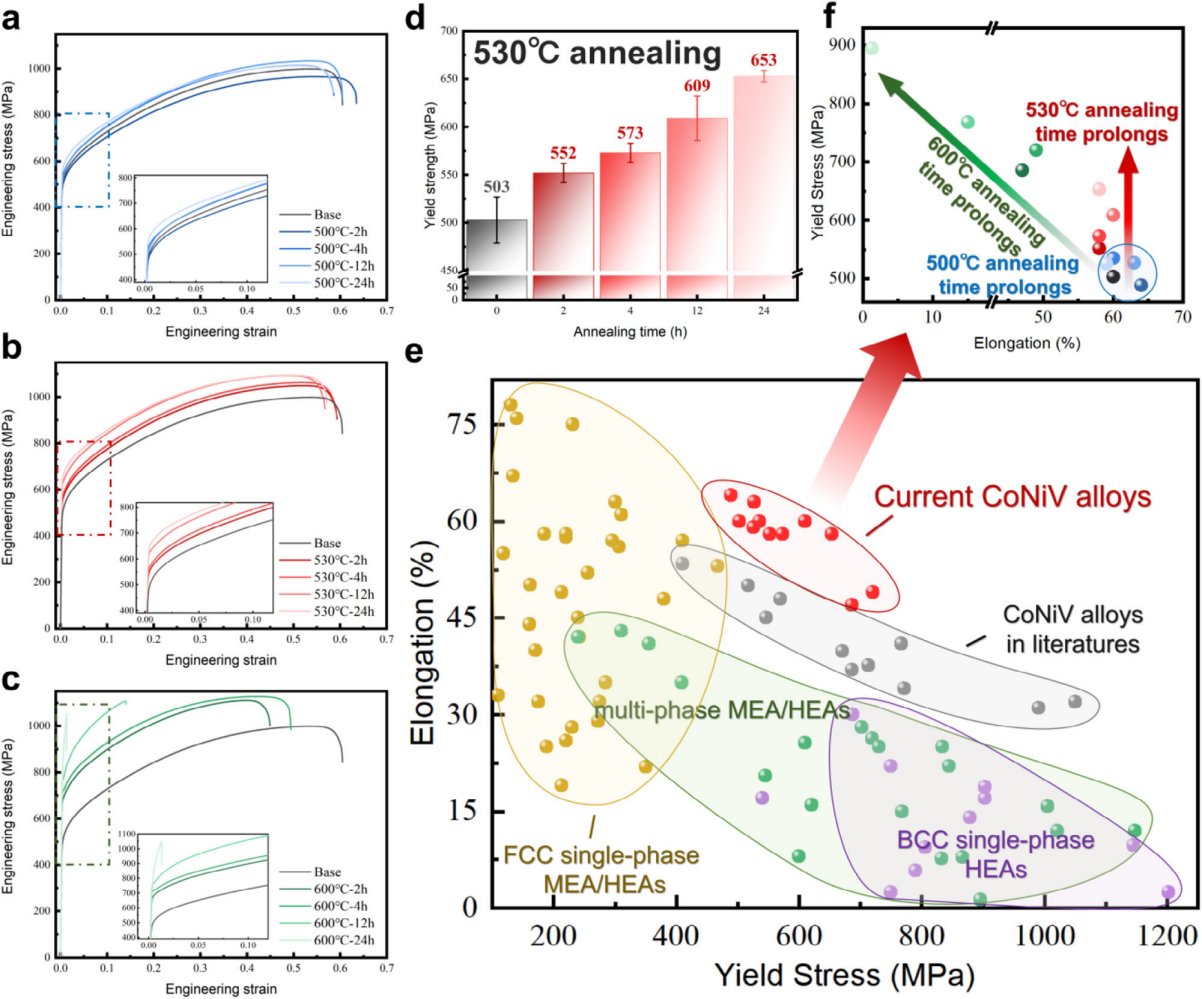
Tensile properties of the CoNiV MEA before and after low‐temperature annealing process. a–c) Engineering stress–strain curves of CoNiV samples annealed at different temperatures (i.e., 500, 530, and 600 °C) for different durations (i.e., 2, 4, 12 and 24 h), respectively. The inset shows the enlarged portions highlighted by red dash lines. d) The yield strength values of CoNiV samples as a function of 530 °C annealing time. e) Overview of yield stress versus elongation for the current CoNiV samples, in comparison with several single or multiphase M/HEAs (see Table S1, Supporting Information for detailed data). The unique combination of strength and ductility of the CoNiV MEA after annealing surpasses that of most other M/HEAs. f) The detailed strengthening effect with different annealing time for the current CoNiV samples.

**Figure** **1**a–c depicts engineering stress–strain curves of CoNiV samples annealed at different temperatures (i.e., 500, 530, and 600 °C) for different durations (i.e., 2, 4, 12, and 24 h), respectively. For comparison, the data for the as‐crystallized sample with no annealing are also included. Surprisingly, the yield strength of the CoNiV samples increases as the low temperature annealing (i.e., annealing at 500 and 530 °C) proceeds, while the ductility keeps almost unchanged. To better clarify this finding, Figure 1d shows the yield strength (σ_y_) values of the CoNiV samples annealed at 530 °C as a function of annealing time. As can be seen, prolonging the annealing time at 530 °C gradually enhances the yield and fracture strength without compromising plasticity. Specifically, compared with that of the as‐recrystallized samples (i.e., 503 MPa), the yield strength increased by ≈30% to 653 MPa after annealing at 530 °C for 24 h, while maintaining high ductility of ∼60%. Nevertheless, it is worthy noting that, although relatively high temperature annealing (i.e., 600 °C) can enhance the strength more significantly, the ductility is dramatically reduced (Figure 1c).

Figure 1e shows yield stress versus elongation for the current CoNiV MEA samples, in comparison with representative single‐ or multi‐phase CCAs reported in literature. Figure 1f highlights the different strengthening effects of different annealing temperatures for the current CoNiV samples. It is evident that low‐temperature annealing (i.e., below 530 °C) enables CoNiV to overcome the trade‐off between strength and ductility, achieving a unique balance between strength and ductility. It is important to note that the selection of temperature and duration for annealing process is critical. In the case of CoNiV MEA, optimal mechanical properties were obtained through low‐temperature annealing treatment at 530 °C for 24 h.

### 2.2 Development of Chemical SROs

In order to elucidate the abnormal enhancement in mechanical properties observed in CoNiV after low‐temperature annealing, we conducted comprehensive characterization and analysis of the microstructures involved. Figure S1a (Supporting Information) shows XRD patterns of CoNiV samples annealed at 530 °C for various annealing durations, indicating that all specimens possess a single‐phase fcc lattice. For clarity, we hereafter designate the recrystallized CoNiV MEA as RC, and the CoNiV MEA annealed at 530 °C for 24 h as AL. EBSD characterization revealed a homogeneous and fully recrystallized microstructure with random grain orientations in both RC and AL, as representatively depicted in Figure S1b,c (Supporting Information). After annealing, the average grain size of CoNiV remained virtually unchanged (Figure S2a, Supporting Information), and the relationship between yield strength and grain size deviated from the Hall‐Petch relationship^[^ ^9^ ^]^ (Figure S2b, Supporting Information), implying that grain boundary strengthening was not a significant contributor to the enhanced yield strength, which will be discussed in detail later.

**Figure 2:**
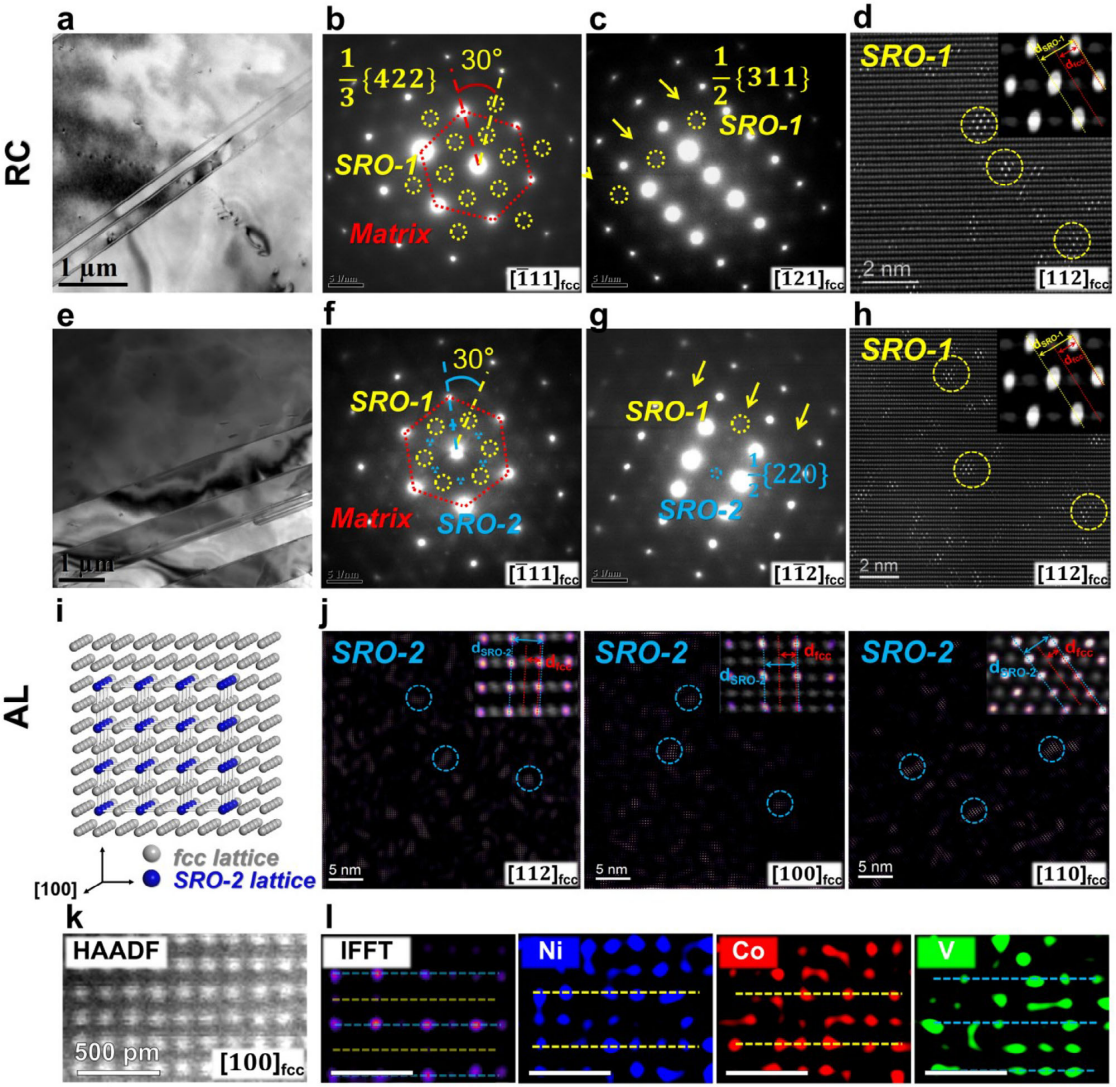
Multi‐scale SROs in CoNiV. a,e) Bright‐field TEM images of RC and AL, respectively. b,c) The SAED patterns of RC along the $\left[\bar{1}11\right]$ and $\left[\bar{1}21\right]$ z.a., respectively. Arrays of extra and diffuse disks (indicated by yellow arrows) appearing at 1/3{422} and 1/2{311} positions (examples are inside the yellow circle) along the $\left[\bar{1}11]\;\mathrm{and}\;[\bar{1}21\right]$ z.a., respectively, indicating existence of SRO‐1 in RC. f,g) Extra disks appearing at the 1/2{220} positions along the $\left[\bar{1}11\right]$ z.a. and the 1/2{220} positions along the $\left[1\bar{1}2\right]$ z.a., respectively (examples are inside the blue circle), indicating coexistence of SRO‐1 and a new SRO (marked as SRO‐2) in AL. d,h) IFFT images of RC and AL, respectively, revealing the illumination of SRO‐1 regions (circled in yellow). Detailed views obtained by overlaying the illuminated SRO‐1 regions with the corresponding fcc lattice in the inset. *d_fcc_* denotes the spacing of different planes in the normal fcc lattice, whereas *d*_*SRO* − 1_ displays the spacing corresponding to the SRO‐1 lattice. j) IFFT images of AL, revealing the illumination of SRO‐2 regions (circled in blue) along the [112], [100] and [110] zone axes, respectively, with the detailed views obtained by overlaying the illuminated ordered regions with the corresponding fcc lattice in the inset. *d_fcc_* denotes the spacing of different planes in the normal fcc lattice, whereas *d*_*SRO* − 2_ displays the spacing corresponding to the SRO‐2 lattice. i) The 3D atomic configuration of a typical SRO‐2 region in fcc lattice. k) The lattice images along the [100] z.a. in AL. l) The lighting‐up position of a typical SRO‐2 region was acquired from the corresponding IFFT image, with the corresponding EDS maps of three components in AL, respectively. Dashed lines reflect the elemental occupation of SRO‐2, namely yellow for V‐depleted planes while blue for V‐enriched planes.

To uncover the underlying mechanism of the unusual hardening effect induced by low‐temperature annealing in CoNiV, further characterization and comparative analysis are conducted. Bright field TEM observations of RC and AL, as shown in **Figure** **2**a,e, respectively, revealed a single‐phase microstructure with annealed twins. Figure 2b,c show the selected area electron diffraction (SAED) patterns of RC along the [$\bar{1}11$], [$\bar{1}21$] z.a., respectively. In addition to a set of sharp diffraction spots from the fcc matrix, highly diffuse but visible extra spots appeared along the [$\bar{1}21$] z.a. at the 1/2{311} positions and the [$\bar{1}11$] z.a. at the 1/3{422} of the fcc lattice in RC. The presence of highly diffused spots indicates that local structures already existed in RC, which corresponds to the previously reported SROs in CoNiV alloys.^[^ ^14^, ^21^ ^]^ Hereafter, we denoted these local structures as SRO‐1. In contrast, AL exhibited a new set of diffuse reflections along different zone axes (Figure 2f,g), in addition to the presence of SRO‐1. These reflections occupy the 1/2{220} positions along the [$\bar{1}11$] z.a. and the 1/2{220} positions along the [$1\bar{1}2$] z.a., respectively, indicating an L1_2_ ordered‐like local structure. Compared with SRO‐1, these new diffraction spots exhibited higher intensity and sharper features. Therefore, it is reasonable to infer that after low‐temperature annealing, a new kind of local atomic structures emerged in CoNiV, while the SRO‐1 structure remains unchanged.

Recent studies have suggested that not only SROs can introduce additional superlattice scattering, but also planar defects (stacking faults) and higher‐order Laue zone diffraction can introduce extra superlattice.^[^ ^22^, ^23^ ^]^ Therefore, to further investigate the SROs in CoNiV after low‐temperature annealing, aberration‐corrected atomic‐resolution high angle annular dark field scanning transmission electron microscope (HAADF–STEM) was used in both reciprocal and true spaces. Based on the lattice images acquired from multiple zone axes for RC and AL (Figure S3, Supporting Information), it was found that the specimens exhibited uniform contrast along all zone axes at different magnifications, and no phase boundaries, ordered structures, or planar defects were found in true space. By utilizing the lattice images and the corresponding additional diffuse reflections from the Fast Fourier Transform (FFT) patterns, inverse FFT (IFFT) images were generated to display the illumination of SRO‐1 regions of RC and AL, as shown in Figure 2d,h, respectively, accompanied by detailed views in the inset. These findings verify that the SRO‐1 is indeed chemical SRO regions.

As mentioned earlier, under appropriate treatments, namely in this case, after low‐temperature annealing, local atomic structures can be manipulated in the alloy system. Figure 2j illustrates atomic‐scale structures of the second local atomic structure (denoted as SRO‐2 hereafter) in AL along the [112], [100] and [110] z.a., respectively, accompanied by detailed views in the inset. Figure 2i depicts a 3D atomic configuration of a typical region of SRO‐2 in the fcc lattice. The motif of SRO‐2 reveals an L1_2_‐type ordered structure that is spherical in 3D space, which can also explain the equivalent intensity of diffuse scattering signals along multiple zone axes (Figure 2f,g).

To explore the influence of chemical compatibility on the development of new local chemical structures in CoNiV, it is necessary to clarify the elemental distribution in SRO‐2. Lattice images and the corresponding EDS mappings were captured for AL along the [100] zone axis. Figure 2k shows the lattice image, including a typical SRO‐2 region (illuminated in the IFFT map) and close‐up views exhibiting preferential locations of the three elements in this region (Figure 2l). It can be observed that SRO‐2 can be best described in terms of V occupancy, specifically three V‐enriched planes (dashed blue lines) sandwiching two V‐depleted planes (in either the Co or the Ni map, under dashed yellow lines). Combining with the atomic configuration given in Figure 2i, it can be inferred that vanadium occupies the corners of the L1_2_ lattice while nickel and cobalt reside at the center of the SRO‐2 lattice.

It was reported that the elemental preference of chemical SROs in CoNiV (i.e., SRO‐1) is predominantly V‐enriched, as evidenced by EDS maps obtained previously.^[^ ^14^, ^21^ ^]^ High‐temperature annealing, such as 800–900 °C, typically leads to the formation of brittle intermetallic phases within the fcc interior or along grain boundaries, specifically κ phase ((Co,Ni)_3_V).^[^ ^24^, ^25^ ^]^ Interestingly, low‐temperature annealing, known as moderate thermal activation, initiates atomic diffusion within the system and triggers a disorder‐order transition characterized by the appearance of SRO‐2 without inducing brittle intermetallic precipitates. The ordered structure of SRO‐2 belongs to an L1_2_‐type, similar to a variant of the Ni_3_V intermetallic compound.^[^ ^26^ ^]^ Due to the structural stability of SROs and the presence of a V‐poor fcc lattice, atomic diffusion in these regions is constrained after low‐temperature annealing, leading to highly dispersed and localized disorder‐order transitions at the atomic scale and eventually giving rise to the appearance of SRO‐2.

### 2.3 Effect of Low‐Temperature Annealing on SRO Development

As previously discussed, annealing processes conducted below the recrystallization threshold are referred to as low‐temperature annealing. Conventionally, such annealing is expected to result in certain degree of material softening. However, our study on CoNiV medium entropy alloy (MEA) challenges this conventional wisdom. As shown in Figure 1, regardless of the annealing temperature (i.e., 500, 530, or 600 °C), an improvement in strength was consistently observed. Notably, annealing at 530°C achieved a significant increase in strength (≈30%) without compromising ductility. This underscores the critical role of selecting an appropriate annealing temperature for the development of the desired SROs.

**Figure 3:**
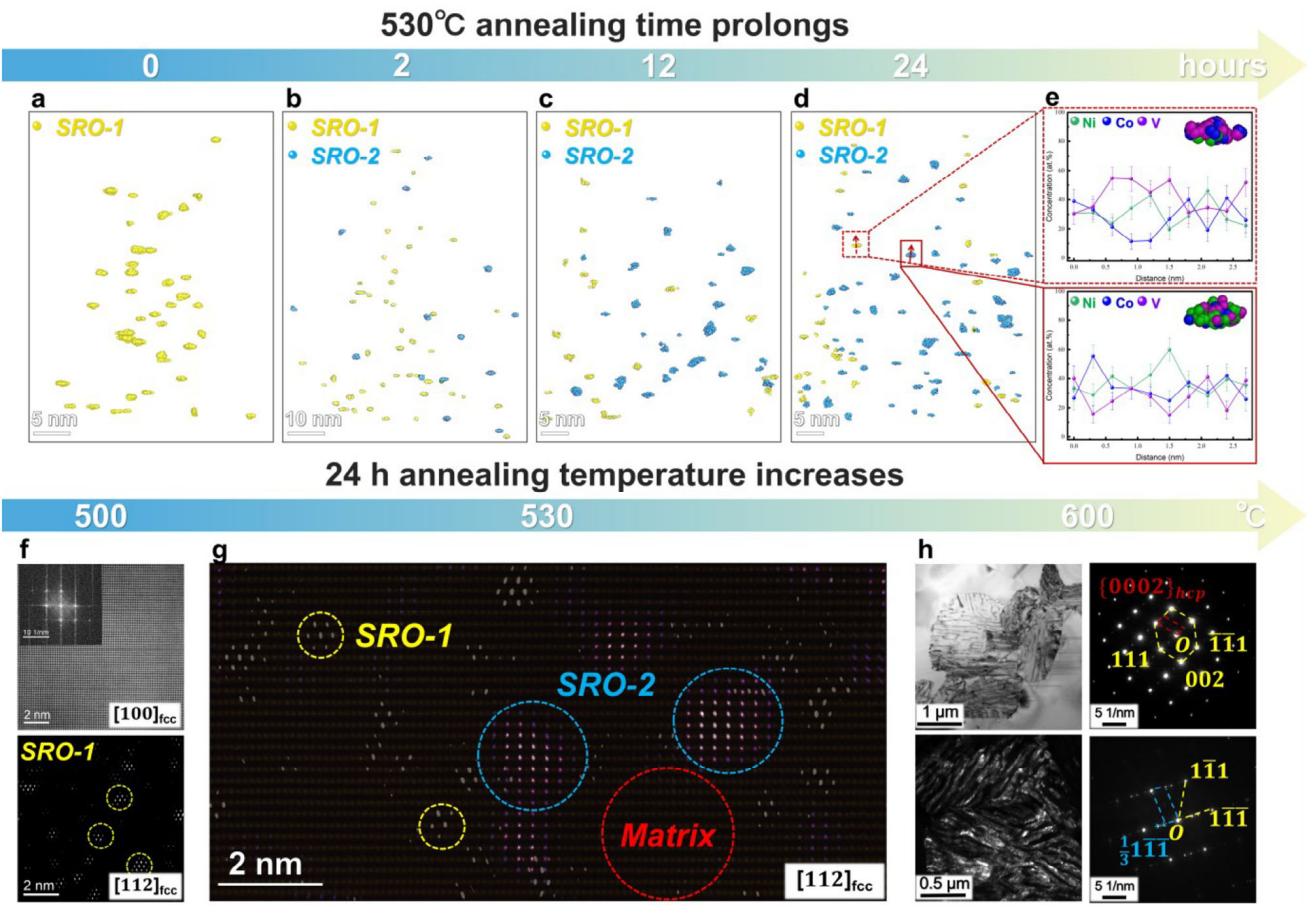
Development of multi‐scaled SROs under different low‐temperature annealing processes. a–d) The 3D reconstruction of multi‐scaled SROs obtained via cluster analysis with 530 °C annealing time prolonging in CoNiV MEAs. Yellow and blue colors correspond to the SRO‐1 and SRO‐2 clusters, respectively. e) The corresponding 1D concentration profiles across typical SRO‐1 and SRO‐2 (red solid and dashed arrows in (d), with the detailed reconstruction of clusters in the inset, respectively. Green, blue and purple colors correspond to Ni, Co, and V atoms, respectively. f–h) The multi‐scaled microstructure evolution with annealing temperature increasing in CoNiV MEAs. f) Atomic structures of CoNiV MEA annealing at 500 °C along the [100] and [112] z.a., respectively, revealing the existence of SRO‐1 while the absence of SRO‐2. g) Atomic structures of CoNiV MEA annealing at 530 °C obtained at the same position along the [112] z.a., with the lighting‐up positions of SRO‐1 regions circled in yellow, SRO‐2 regions circled in blue, and matrix region circled in red. h) Microstructures of CoNiV MEA annealing at 600 °C along the [110] and [111] z.a., respectively, revealing emergence of HCP phase.

Next, we will delve into the development of multi‐scaled SROs under various low‐temperature annealing processes. By annealing CoNiV samples at 530 °C for different durations (i.e., 2, 4, 12, and 24 h), respectively, the evolution of both SRO‐1 and SRO‐2 can be visually observed through 3D reconstruction profiles using cluster analysis, as depicted in **Figure** **3**a–d. This analysis was based on the data obtained from APT measurements (Figure S4, Supporting Information), and the evolution of SROs was quantified and listed in Figure S5 (Supporting Information). As the annealing time prolongs, SRO‐2 regions were directly formed from the fcc matrix, rather than from pre‐existing SRO‐1 regions. Moreover, extended annealing increases the number density of SRO‐2 without significant growth in size (see Figure S7e, Supporting Information). This behavior contrasts with conventional nanoscale precipitate formation, which typically involves a nucleation and growth process, revealing a unique formation mechanism specific to the SROs. Elemental partitioning characteristics, as evidenced by the reconstructed clusters in Figure 3e, demonstrate that SRO‐1 is predominantly V‐rich while SRO‐2 is Ni enrichment. This is consistent with the EDS maps (^[^ ^14^, ^21^ ^]^ and Figure 2). The structural stability of SRO‐1, coupled with the V‐deficient fcc matrix, significantly limits atomic diffusion during 530 °C annealing. These constrained diffusion conditions facilitate localized disorder‐order transitions at the atomic scale, ultimately leading to the highly dispersed formation of SRO‐2.

The microstructural evolution of CoNiV samples annealed at 500, 530, and 600 °C for 24 h is presented in Figure 3f–h. Annealing at 500 °C exclusively yields SRO‐1 without SRO‐2 formation, resulting in a marginal increase in yield strength from 503 to 526 MPa (see Figure 1a). This temperature is insufficient for SRO‐2 development. In contrast, annealing at 600 °C triggers the formation of brittle hexagonal close‐packed (HCP) phases, leading to a substantial yield strength enhancement (503 to 895 MPa) but a catastrophic loss of ductility (Figure 1c, from 60% to 0.8%). This temperature‐dependent phase transformation, consistent with DSC (differential scanning calorimetry) results shown in Figure S6 (Supporting Information), demonstrates that temperatures ≥600 °C are unsuitable for SRO formation due to phase stability constraints. The annealing treatment at 530 °C proves particularly significant as it facilitates the formation of SRO‐2 while maintaining SRO‐1 (as depicted in Figure 3g), leading to an exceptional balance between strength and ductility. These findings highlight the critical importance of precise control of annealing temperature in tailoring atomic‐scale structures and optimizing mechanical properties in CCAs.

Interestingly, such low‐temperature hardening effect is not significant in the similar CoNiCr MEA due to the limitation on SRO development. For comparative analysis, a parallel investigation was conducted on CoNiCr (i.e. produced under same processing conditions), and their microstructures are shown in Figure S7 (Supporting Information). All the samples still maintained a single‐phase fcc structure after annealing, along with fully recrystallized microstructures exhibiting randomly distributed grain orientations. Meanwhile, we also obtained bright field images and the corresponding SAED patterns of the as‐recrystallized and the specimen annealed at 530 °C for 24 h along different zone axes (Figure S7f–j, Supporting Information). A single type of SROs was consistently observed before and after low‐temperature annealing, as evidenced by the diffuse reflections observed along [111] z.a. Due to the inability to induce new chemical SROs in CoNiCr, the pre‐existing SROs primarily composed of the previously reported CSROs^[^ ^13^, ^27^ ^]^ exhibit high structural stability. As a result, very marginal increase in the yield strength was observed in a similar fcc CoNiCr MEA after low temperature annealing, although the ductility kept almost unchanged (Figures S8 and S9, Supporting Information). Both CoNiV and CoNiCr are single‐phase fcc solid solutions with identical configurational entropy, however, the primary distinction lies in their constituent elements, specifically the difference between V and Cr, which leads to a notable disparity in chemical compatibility. Generally speaking, the formation of SROs in CCAs is thermodynamically and kinetically favorable due to reduction in the Gibbs free energy of the system.^[^ ^28^ ^]^ By comparing two MEA systems studied here, however, it is clear that not all alloy systems can induce formation of new SROs through low‐temperature annealing. Compared with Cr, vanadium has a larger atomic size difference with Ni and Co, and larger negative mixing enthalpy. As a result, it has strong tendency to form ordered structures with Ni and/or Co.^[^ ^29^ ^]^ Therefore, formation and stability of SROs in CCAs are closely related to chemical compatibility of their constituent elements, such as atomic size and mixing enthalpy difference.

### 2.4 SRO‐Mediated Abnormal Hardening

The 530 °C@24h annealing treatment has been identified as the optimal condition for CoNiV samples, as it effectively induces the formation of SRO‐2 and is considered the most favorable for its development. In this section, the underlying mechanism of how SRO‐2 formation contributes to the superior mechanical performance, in contrast to the limited effect of SRO‐1, will be thoroughly discussed. Additionally, we explore how SROs, particularly SRO‐2, influence deformation behavior of the alloy.

First, we estimated the strengthening contributions from various mechanisms in RC and AL (analyzed in Experimental Section), as summarized in Figure S10a (Supporting Information). For RC, the reinforcement effect of SROs is entirely attributed to SRO‐1, contributing ≈53 MPa to the yield strength. In contrast, for AL, the strengthening effect of SROs comes from both SRO‐1 and SRO‐2, contributing ≈58 and 123 MPa to the yield strength, respectively. It is evident that the predicted data exhibit reasonable consistency with the experimental values. Most of SRO‐1 has a thickness of mere 3–5 atomic layer and a diffusive interface with the matrix, consistent with previous reports.^[^ ^14^ ^]^ Its volume fraction remains nearly constant (16% to 15% of the total area) with a slight increase in size (from 0.78 to 1.02 nm) during annealing (see Figure S10b, Supporting Information), indicating its high structural stability. In contrast, SRO‐2 was easily stimulated at low temperatures and gradually developed an average size of 1.64 ± 0.39 nm and a volume fraction constituting 20% ± 6.3% of the total area, covering ≈10 atom layers. These larger clusters could be more effective in providing exceptional hardening.

**Figure 4:**
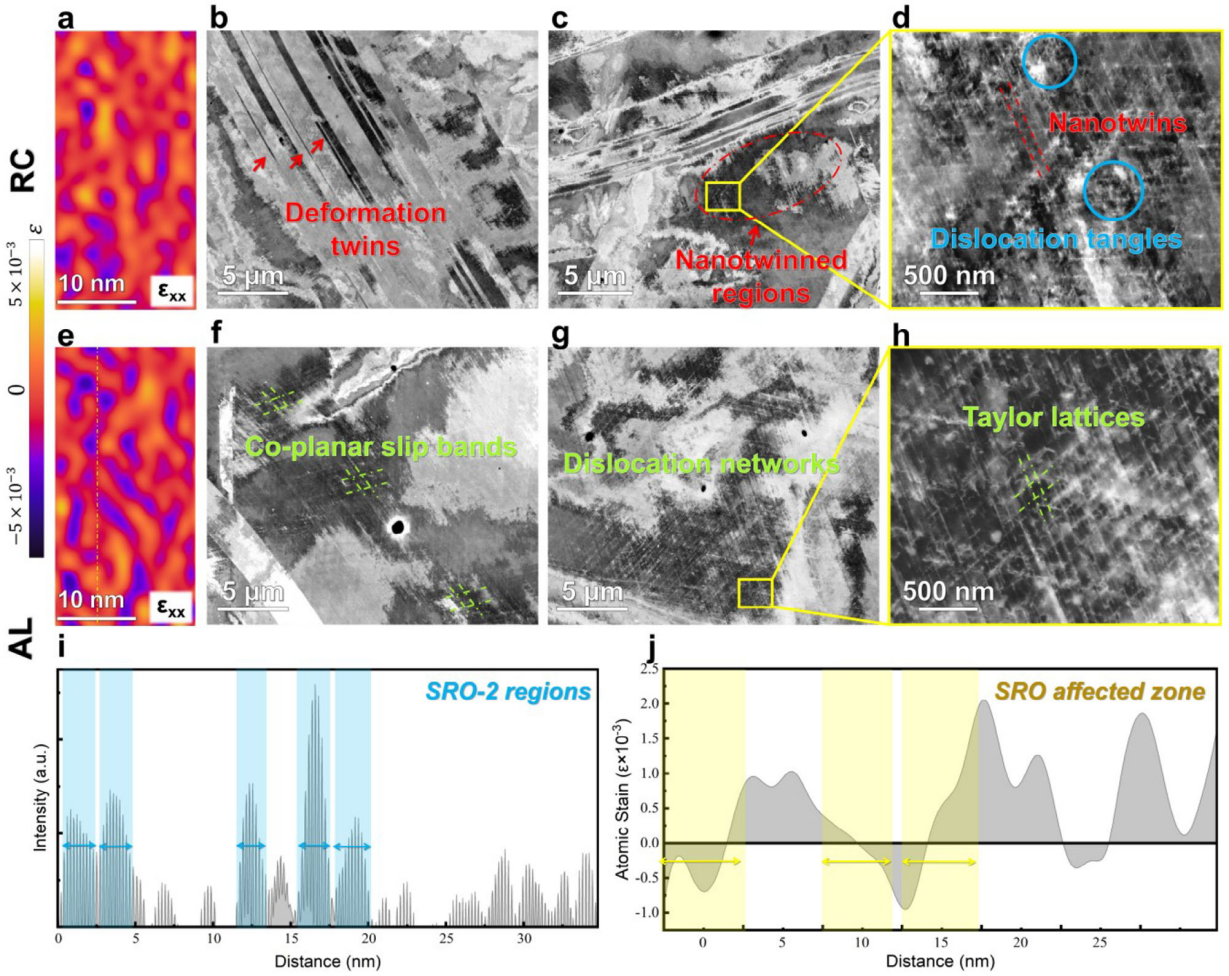
Deformation mechanisms of CoNiV. a,e) Atomic‐strain mapping (ε_xx,_ horizontal normal strain) of RC and AL along the [112] z.a. before tensile deformation, respectively. b–d) Typical deformation structures in RC after tensile fracture, with the deformation twins/nanotwinned regions arrowed in red and dislocation tangles circled in blue, respectively. f–h) Typical deformation structures in AL after tensile fracture, with the co‐planar slip bands/Taylor lattices highlighted in green. d,h) The enlarged images of the yellow boxed regions in (c) and (g), respectively. i) The additional diffraction intensities of the line scan in (e). j) Statistical distribution for the corresponding atomic strain of the line scan in (e).

To investigate how SRO‐2 formation influences the deformation behavior of CoNiV, we performed strain field analysis on RC and AL samples before deformation using the Geometric phase analysis (GPA) method. **Figure** **4**a,e depicts the atomic‐strain mappings of RC and AL along the [112] z.a., respectively. From the mapping of ε_xx_ (Figure 4a,e, horizontal normal strain), ε_xy_ and ε_yy_ (Figure S11, Supporting Information shear strain, and vertical normal strain, respectively), it can be observed that compared to RC, AL always exhibit greater fluctuations in atomic strains, indicating that the presence of SRO‐2 disrupts the surrounding strain field. Subsequently, a line scan depicted in Figure 4e reveals additional diffraction intensities (as illustrated in Figure 4i) and statistical distribution of the corresponding atomic strain (as shown in Figure 4j). SRO‐2 induces an affected zone in the stress field, slightly larger than its own size, characterized by a reversal of positive/negative strains (tensile/compressive strains). By highlighting the positions of SRO‐2, we observed a transition in strain from positive to negative (or vice versa) influenced by SRO‐2, indicating that SROs induced an affected zone in the stress field, which is slightly larger than its own size. These affected zones manifest as a reversal of positive/negative strains (i.e., tensile/compressive strains).

To delve into the deformation mechanism of CoNiV with/without SRO‐2, we further scrutinized the microstructures of RC and AL after tensile fracture using electron channel contrast imaging (ECCI). Figure 4b–d illustrates typical deformation structures in RC, while Figure 4f–h depicts those in AL. All the images were obtained from multiple fields of view. Generally speaking, deformation mechanisms in metallic materials are fundamentally governed by the relationship between the applied flow stress and the critical stress required to activate various deformation carriers such as dislocations, SFs and twins. During loading, numerous deformation twins with sizes ranging from nanometers to micrometers were observed in RC (Figure 4b–d), consistent with previous experimental observations and simulation results.^[^ ^30^, ^31^ ^]^ As previously reported,^[^ ^32^ ^]^ this phenomenon is attributed to a low stacking fault energy (SFE) of ≈30 mJ · m^2^. Figure 4d presents an enlarged image of the yellow boxed region in Figure 4c, revealing significant dislocation entanglement in RC due to interaction between pre‐existing chemical SROs and moving dislocations.^[^ ^14^ ^]^ After annealing at 530 °C, the deformation mechanism of CoNiV underwent a remarkable evolution, as evidenced by changes in the primary deformation carriers. Specifically, deformation twinning was no longer observed in AL, and instead replaced by extensive co‐planar slip bands. These slip bands are comprised of a dense network of dislocations known as Taylor lattices,^[^ ^33^ ^]^ which originate from the planar slip of dislocations, as circled in Figure 4f–h. It is worth noting that these co‐planar slip bands typically extend over tens of microns with minimal dislocation pile‐ups or tangles unless they encounter interfaces such as grain boundaries or annealed twin boundaries. This shift in deformation mechanism between the RC and AL samples can be attributed to the competitive interplay between the SRO and SFE in governing plastic deformation.^[^ ^34^ ^]^ Recent studies have demonstrated that SROs induce spatial fluctuations in the SFE landscape, which is strongly correlated with local chemical inhomogeneities.^[^ ^35^ ^]^ In RC, where only SRO‐1 is present with a limited volume fraction (∼16%), deformation resulted in the formation of numerous nanotwins. This observation indicates that the SRO concentration is insufficient to suppress the formation of either SFs or nanotwins. In contrast, the AL sample exhibits a dual‐SRO configuration (i.e., coexistence of SRO‐1 and SRO‐2) with a total SRO volume fraction of ∼35%. Notably, deformation twins are entirely absent in this case, replaced by extensive co‐planar slip bands. This transition suggests that the emergence and development of SRO‐2 not only increases the overall SRO concentration but also introduces additional modulations to the SFE landscape. These combined effects effectively suppress twin nucleation and promote planar slip activity, highlighting the pivotal role of SRO evolution in dictating deformation mechanisms in M/HEAs.

According to previous studies, the presence of SROs in fcc CCAs introduces a rugged energy landscape that affects atomic arrangement and dislocation motion paths.^[^ ^13^, ^14^, ^35^ ^]^ In CoNiV, due to the sub‐nanometer scale and diffuse distribution of pre‐existing SRO‐1, dislocations inevitably meet SROs during movement, and generate certain pinning and hindering effects, leading to dislocation entanglement, extensive dislocation pile‐up, and multipole structures. Low‐temperature annealing promotes the formation of new SROs (i.e., SRO‐2), which exhibit similar dispersion but larger size compared to SRO‐1. Based on the distribution of atomic strains, it is known that SRO‐2 enhanced the fluctuation of atomic strains and induced the reversal of positive/negative strains. All these characteristics make SRO‐2 gentle obstacles that prompt dislocations to seek new pathways for minimizing energy penalties. As a result, this behavior enhanced the planarity of dislocation slip, giving rise to large ductility.

## 3 Conclusion

In this study, we have successfully developed a novel strengthening strategy for CoNiV MEA through low‐temperature annealing. This approach resulted in a significant increase in yield strength from 503 to 653 MPa while maintaining a plasticity of ≈60%. The enhancement in mechanical properties is attributed to the development of multi‐scale SROs, which were characterized using advanced experimental techniques.

During low‐temperature annealing, a novel kind of SROs with an L1_2_‐type ordered structure was developed in CoNiV, which typically consist of ≈10 layers of atoms and are homogenously dispersed with a volume fraction of ≈20% in the fcc lattice. The development of SROs in CoNiV induced an affected zone, intensifying atomic strain fluctuations and causing a reversal of positive/negative strains. This effect transformed SROs into gentle obstacles that prompt dislocations to seek new pathways to minimize energy penalties, effectively enhancing strength of the material.

Moreover, the development of SROs promoted the planarity of dislocation slip and reduced dislocation entanglement, thereby maintaining large ductility and work‐hardening capability. Our findings establish a robust link between the intrinsic structural features of H/MEAs and their unique mechanical properties. This study demonstrates the potential for using SROs as genetic characteristics for alloy design, opening new avenues for future development of CCAs in high‐performance structural applications.

## 4 Experimental Section

### Sample Preparation and Mechanical Property Tests

Ingots with equiatomic ratios of CoNiV MEAs were prepared by arc‐melting of a mixture of pure metals (purity ≥ 99.9 wt.%) under argon atmosphere. The ingots were remelted at least six times to ensure compositional homogenization. Subsequently, the ingots were homogenized at 1200 °C for 24 h, followed by cold‐rolling to achieve a thickness reduction of 90%. To minimize variations of sample grain size and reduce differences in grain boundary strengthening, the cold‐rolled sheets were recrystallized at 1000 °C for 30 min, following by water‐quenching. The sheets were then annealed at temperatures of 530°C for varying durations (0 min, 2 h, 4 h,12 h, and 24 h), followed by water quenching.

Dog‐bone‐shaped tensile specimens with nominal gauge dimensions of 20 mm (length) × 5 mm (width) × 1.3 mm (thickness) were extracted from rectangular plates using electrical discharge machining. The specimens were subsequently polished with 2000‐grit SiC paper, and strain measurements were conducted using a 15‐mm gauge length extensometer. A CMT4105 universal electronic tensile testing machine was employed for tensile tests at room temperature with a nominal strain rate of 1 × 10^−3^ s^−1^.

### Microstructural Characterization

Phase constitution of all samples was verified using an X‐ray diffractometer (XRD, Rigaku‐Smart Lab 9 kW) with a Cu target (Cu Kα radiation) operated at a voltage of 45 kV and a current of 200 mA. The scanning angle ranged from 30° to 100°, with a scanning speed of 10° min^−1^. Electron backscatter diffraction (EBSD) experiments was performed at an accelerating voltage of 20 kV in a Zeiss GEMINI450 SEM (scanning electron microscope) equipped with an Oxford EBSD camera. EBSD maps were collected using a step size of 0.5 µm, and the data was analyzed using AZtecCrystal software.^[^ ^36^ ^]^ Microstructural evolutions in the deformed samples were examined by electron channel contrast imaging (ECCI, Zeiss GEMINI450), with an accelerating voltage of 25 kV and an electric current of 2.5 nA. The ECCI samples were initially ground using 2000‐grit SiC papers and subsequently subjected to mechanical polishing in a metallographic polishing machine (Qpol Vibro).

For TEM (transmission electron microscope) observation, specimens were first mechanically ground to 50 µm thickness, and then twin‐jet electropolished using HClO_4_ (10%) and C_2_H_5_OH (90%) solution at approximately −30 °C and 25V/45mA. TEM characterization was conducted in an FEI Tecnai 30 microscope at 300 kV. Atomic‐resolution TEM and HAADF‐STEM experiments were performed on an aberration‐corrected STEM (FEI Titan Cubed Themis G2 300) operated at 300 kV to analyze atomic structure of the MEAs. The convergence semi‐angle for imaging is 30 mrad, and the collection semi‐angle snap is 39 to 200 mrad for the HAADF imaging. Geometric phase analysis (GPA) was performed to investigate the distribution of strain fields using the HAADF images. Quantitative energy dispersive X‐ray spectroscopy (EDS) mappings with atomic‐resolution were conducted in an FEI Spectra 300 microscope at 300 kV to analyze elemental distribution of the MEAs.

Sharp tip specimens for atom probe tomography (APT) were prepared by focused ion beam milling on a dual‐beam FEI Helios 60040. Atom probe tomography and 3D elemental distribution analyses were carried out in CAMECA Instruments LEAP 5000XR local electrode atom probe system. The specimens were analyzed in laser mode under an ultrahigh vacuum of ≈2.5 × 10^−11^ Torr at 50 K, and the pulse frequency and pulse fractions were 250 kHz and 20%, respectively. The CAMECA integrated visualization and analysis software IVAS 3.8.4 was used for data processing and 3D atomic reconstruction.

### Strengthening Mechanisms Analysis

To understand the strengthening behavior in CoNiV before and after low‐temperature annealing, here analyze the strengthening mechanism below. The yield strength, representing the resistance to flow observed at the onset of plastic deformation, is determined by summing up three distinct contributions applicable to our specific case: 1) intrinsic friction stress, 2) grain‐boundary strengthening (known as Hall‐Petch effect), and 3) SRO strengthening. It should be noted that the materials used in the study possess fully‐recrystallized structures with a very low dislocation density values of 10^11^–10^12^ m^−2^,^[^ ^37^ ^]^ rendering dislocation hardening negligible. Furthermore, there are no precipitates in our MEAs has been demonstrated, thereby ruling out any consideration of precipitation hardening. Therefore, the yield strength can be expressed as:

(1) $$\sigma_{y}=\sigma_{0}+\Delta \sigma_{GB}+\Delta \sigma_{SRO}$$

where σ_0_ is the intrinsic strength, or the so‐called lattice friction stress, which can be expressed using the following equation^[^ ^38^, ^39^ ^]^:

(2) $$\sigma_{0}=2MG/\left(1-\nu \right)\cdot \exp\left(-2\pi w/b\right)$$

where *M*  =  3.06 is the Taylor factor for fcc, *G*  =  72 *GPa* is the shear modulus, ν  =  0.334 is the Poisson's ratio, *w*/*b*  =  1.19 is the ratio of the dislocation width and the magnitude of the Burger vector for the CoNiV MEA.^[^ ^9^ ^]^ According to Equation (2), the intrinsic strength of CoNiV MEA is determined as 374 MPa.

Δσ_*GB*_ is the strengthening contribution from grain boundaries according to the Hall‐Petch relationships:

(3) $$\Delta \;\sigma_{GB}=k_{\gamma }\cdot d^{-1/2}$$

where *k*_γ_ =  864 *MPa* · µ*m* ^1/2^ is the Hall‐Petch coefficient of CoNiV MEA acquired from Ref.[9], and the values of the average grain size *d* which were derived from the EBSD maps in Figure S1 (Supporting Information). Thus, Δ σ_*GB* − *RC*_ =  144 *MPa* and Δ σ_*GB* − *AL*_ =  145 *MPa* were obtained.

Next, considering the aforementioned characteristics, the nanoparticle shearing mechanism, in traditional alloys, was adopted to elucidate three factors that account for the strengthening effects of multi‐scale SROs^[^ ^40^, ^41^, ^42^ ^]^: coherency strengthening (Δσ_*c*_) resulted from SRO‐matrix coherency, modulus strengthening (Δσ_*m*_) caused by modulus mismatch, and ordering strengthening (Δσ_*o*_) induced by disorder‐order transition. The equations representing these contributions are as follows^[^ ^43^ ^]^:

(4) $$\Delta \sigma_{o}=M\cdot 0.81\frac{\gamma_{APB}}{2b}{\left(\frac{3\pi f}{8}\right)}^{1/2}$$

(5) $$\Delta \sigma_{c}=M\cdot \alpha_{\varepsilon }{\left(G\cdot \varepsilon \right)}^{3/2}{\left(\frac{rf}{0.5Gb}\right)}^{1/2}$$

(6) $$\Delta \sigma_{m}=M\cdot 0.0055{\left(\Delta G\right)}^{3/2}{\left(\frac{2f}{G}\right)}^{1/2}{\left(\frac{r}{b}\right)}^{\left(3m/2\right)-1}$$

Among them, γ_*APB*_ is the inverse boundary energy of SRO. Due to the lack of literature data, the anti‐phase boundary energy of SROs in CoNiV, i.e. γ_*APB* − *SRO*1_ =  0.12 *J*/*m* ^2^, was adopted from the corresponding data of SROs in CoNiCr MEA^[^ ^13^ ^]^ while γ_*APB* − *SRO*2_ =  0.12 *J*/*m* ^2^ adopted from the corresponding data of Ni_3_Al precipitates in Ni‐based superalloys.^[^ ^44^ ^]^ *m*  =  0.85 is a constant, *f* represents the volume fraction of SROs, which was derived from the IFFT maps, *b*  =  0.255 *nm* is the Burger vector of the matrix.^[^ ^34^ ^]^ In addition, α_ε_ =  2.6 and ε ≈ 2/3 · (Δ*a*/*a*) is the constrained lattice parameter mismatch with Δa/a=∼0.0026 obtained from similar systems.^[^ ^34^ ^]^ *r* is the average SRO size measured from the IFFT maps, Δ*G* is the shear modulus mismatch between the matrix and the SROs, and a rule of mixtures was applied to determine *G*_*SRO* − 1_ =  76 *GPa* and *G*_*SRO* − 2_ =  81*GPa* using *G_NiCo_* =  84 *GPa*.^[^ ^45^, ^46^ ^]^ By substituting all parameters into the above equations, values of ordering strengthening, coherency strengthening and modulus strengthening can be calculated. The contribution of SRO strengthening Δσ_*SRO*_ depends on the larger value between Δσ_*o*_ and Δσ_*c*_ + Δσ_*m*_:

(7) $$\Delta \sigma_{SRO}=max\left\{\Delta \sigma_{o},\Delta \sigma_{c}+\Delta \sigma_{m}\right\}$$

## Conflict of Interest

The authors declare no conflict of interest.

## Author Contributions

Y.W., X.B.Z., and Z.P.L. supervised and conceived this project. Y.H.W. carried out materials fabrication, mechanical and microstructure characterizations. Y.Y., Y.H., and X.Y.H. conducted and analyzed TEM experiments. Y.H.W., Y.W., and Z.P.L. wrote and revised the manuscript. All authors including H.W., X.J.L., and S.H.J. discussed the results and commented on the manuscript.

## Supporting information



Supporting Information

## Data Availability

The data that support the findings of this study are available from the corresponding author upon reasonable request.
