# Supplementary material for: Low‐Temperature Annealing Triggered Abnormal Strengthening in a Complex Concentration Alloy via Evolutive Short‐Range Ordering
Source: Adv Sci (Weinh). 2025 Jul 3;12(35):e06962. doi: 10.1002/advs.202506962 (PMC12463031; doi:10.1002/advs.202506962)
Supplement: Supplementary file 1 — Supporting Information [file ADVS-12-e06962-s001.docx]

Supporting Information

Title

Low-Temperature Annealing Triggered Abnormal Strengthening in a complex concentration alloy via evolutive Short-Range Ordering

**Authors**

*Yihan Wang,^1^ Yuan Wu,^1^* Yong Yu,^1,2^ Yang He,^3^ Xinyang Yu,^3^ Xiongjun Liu,^1^ Hui Wang,^1^ Suihe Jiang,^1^ Xiaobin Zhang,^1^*, Zhaoping Lu,^1^**

**Table S1** Compositions, microstructure, and tensile properties of previously reported M/HEAs.

| **Alloys** | **Phases** | **YS (MPa)** | **UTS (MPa)** | **U. EL (%)** | **Ref.** |
| --- | --- | --- | --- | --- | --- |
| CoCrFeMnNi | FCC | 410 | 763 | 57 | [S1] |
| CoCrNi | FCC | 306 | 766 | 56 | [S2] |
| CoCrNi | FCC | 273 | 757 | 80 | Our work |
| CoCrNi | FCC | 279 | 772 | 73 | Our work |
| CoCrNi | FCC | 275 | 773 | 70 | Our work |
| CoCrNi | FCC | 289 | 779 | 69 | Our work |
| CoCrNi | FCC | 278 | 784 | 75 | Our work |
| CoCrMnNi | FCC | 285 | 574 | 35 | [S2] |
| CoCrFeNi | FCC | 273 | 594 | 29 | [S2] |
| CoMnNi | FCC | 230 | 526 | 28 | [S2] |
| FeMnNi | FCC | 220 | 476 | 26 | [S2] |
| CoFeNi | FCC | 213 | 358 | 19 | [S2] |
| FeNi | FCC | 188 | 358 | 25 | [S2] |
| CoFeMnNi | FCC | 175 | 406 | 32 | [S2] |
| CoNi | FCC | 110 | 391 | 33 | [S2] |
| Ni_80_Mo_20_ | FCC | 1047 | 1301 | 37 | [S3] |
| Ni_80_Mo_20_ | FCC | 929 | 1240 | 40 | [S3] |
| Ni_80_Mo_20_ | FCC | 700 | 1160 | 43 | [S3] |
| Ni_80_Mo_20_ | FCC | 523 | 1078 | 55 | [S3] |
| Al_0.1_CoCrFeNi | FCC | 160 | - | 44 | [S4] |
| Al_0.25_CoCrFeNi | FCC | 118 | - | 55 | [S4] |
| Al_0.3_CoCrFeNi | FCC | 275 | - | 32 | [S4] |
| Al_0.4_CoCrFeMnNi | FCC | 242 | 529 | 42 | [S5] |
| Al_0.7_Cr_0.5_Fe_3.6_Mn_3.1_Ni | FCC | 170 | 375 | 40 | [S6] |
| Al_0.7_Cr_0.5_Fe_3.6_Mn_3.1_NiC_0.09_ | FCC | 380 | 870 | 48 | [S6] |
| Co_1.4_CrFeMnNi | FCC | 586 | 715 | 24.3 | [S7] |
| CoCr_1.3_FeMnNi_0.7_ | FCC | 162 | 462 | 50.1 | [S7] |
| Co_0.19_Cr_0.08_Fe_1.58_Mn_1.08_Ni | FCC | 240 | 645 | 45 | [S8] |
| Co0.1Cr0.1Fe0.4Mn0.4 | FCC | 213 | 471 | 49 | [S9] |
| CoCrFeMo_0.2_Ni | FCC | 255 | 590 | 52 | [S10] |
| Co_1.75_Cr_0.75_FeMo_0.5_Ni | FCC | 350 | 720 | 21.9 | [S11] |
| Co_2.125_Cr_0.625_FeMo_0.25_Ni | FCC | 220 | 540 | 57.5 | [S11] |
| Co_0.32_Cr_0.23_FeNi_0.13_Mo_0.14_ | FCC | 296 | 693 | 57 | [S12] |
| Ni_63.2_V_36.8_ | FCC | 565 | 1031 | 46 | [S3] |
| Al_0.3_CoCrFeNi | FCC+B_2_ | 702 | 1002 | 28 | [S13] |
| Al_0.5_CoCrFeNi | FCC+BCC+B_2_ | 834 | 1220 | 25 | [S14] |
| Al_0.7_CoCrFeNi | FCC+BCC+B_2_ | 600 | 740 | 8 | [S15] |
| AlCoCrFeNi_2.1_ | FCC+B_2_ | 844 | 1175 | 22 | [S16] |
| Al_0.7_CoCrFe_2_Ni | FCC+BCC+B_2_ | 866 | 1223 | 7.9 | [S17] |
| Al_0.2_CoCrCu_0.2_FeNi_2_ | FCC+L1_2_ | 719 | 1048 | 26.3 | [S18] |
| HfNbTiZr | BCC | 879 | 969 | 14 | [S19] |
| HfTaTiZr | BCC | 1356 | 1452 | 2.5 | [S20] |
| HfTa0.6TiZr | BCC | 750 | 1110 | 20 | [S20] |
| HfNbTaTiZr | BCC | 1202 | 1295 | 2.5 | [S21] |
| HfNbTaTiZr | BCC | 1145 | 1262 | 9.7 | [S21] |
| Hf_0.5_Nb_0.5_Ta_0.5_Ti_1.5_Zr | BCC | 903 | 990 | 17 | [S22] |
| Al_0.6_CrFe_2_Mn_1.2_Ni_0.8_ | BCC+B2 | 750 | 880 | 2.5 | [S23] |
| CoNiV | FCC+L2_1_+σ | 1260 | 1590 | 27 | [S4] |
| CoNiV | FCC+L2_1_+σ | 1500 | 1730 | 8 | [S4] |
| CoNiV | FCC+L2_1_+σ | 1050 | 1480 | 32 | [S4] |
| CoNiV | FCC+L2_1_+σ | 570 | 1120 | 48 | [S4] |
| CoNiV | FCC | 517 | 1049 | 50 | [S24] |
| CoNiV | FCC | 767 | 1221 | 41 | [S24] |
| CoNiV | FCC | 991 | 1359 | 31 | [S24] |
| CoNiV | FCC | 771 | 1226 | 34.1 | [S3] |
| CoNiV | FCC | 713 | 1173 | 37.7 | [S3] |
| CoNiV | FCC | 671 | 1142 | 39.8 | [S3] |
| CoNiV | FCC | 410 | 814 | 53.4 | [S3] |
| CoNiV | FCC | 503 | 999 | 60 | Our work |
| CoNiV | FCC | 552 | 1037 | 58 | Our work |
| CoNiV | FCC | 573 | 1063 | 58 | Our work |
| CoNiV | FCC | 609 | 1082 | 60 | Our work |
| CoNiV | FCC | 653 | 1095 | 58 | Our work |
| Al_0.3_CoCrFeNi | FCC+L1_2_ | 310 | 525 | 43 | [S25] |
| Al_0.3_CoCrFeNi | FCC+B_2_ | 240 | 570 | 42 | [S25] |
| Al_0.3_CoCrFeNi | FCC+B_2_ | 702 | 1002 | 28 | [S26] |
| Al_0.3_CoCrFeNi | FCC+B_2_ | 1147 | 1207 | 12 | [S27] |
| Al_0.5_CoCrFeNi | FCC+BCC | 355 | 714 | 41 | [S14] |
| AlCoCrFeNi_2.1_ | FCC+L1_2_+B_2_ | 620 | 1050 | 16 | [S16] |
| AlCoCrFeNi_2.2_ | FCC+B_2_ | 545 | 1120 | 20.5 | [S28] |
| Al_0.17_CoCrFeNiTi_0.09_ | FCC+L1_2_+Ni_2_AlTi | 1005 | 1273 | 15.8 | [S5] |
| Al_0.5_CoCrCuFeNi | 2FCC | 610 | 780 | 25.6 | [S29] |
| Al_0.5_CoCrCuFeNi | 2FCC | 1021 | 1030 | 12 | [S29] |
| Al_0.6_CoCrFeMnNi | FCC+BCC+B_2_ | 832 | 1174 | 7.7 | [S5] |
| Al_0.5_CoCrFeMnNi | FCC+B_2_ | 409 | 755 | 35 | [S30] |
| Al_0.5_CoCrFeMnNi | FCC+B_2_ | 730 | 968 | 25 | [S30] |


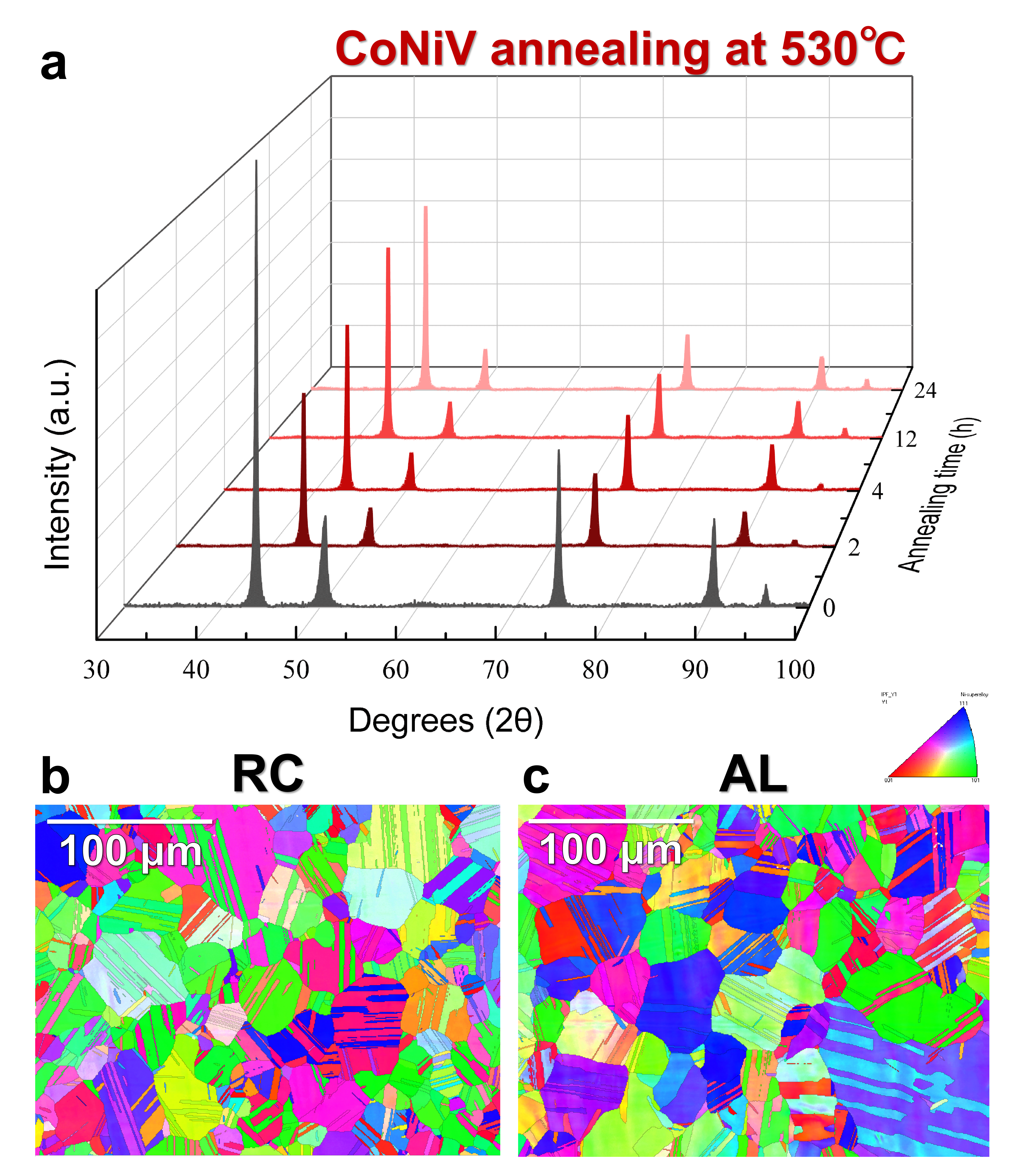


**Figure S1.** Structure and phase constitution of the CoNiV MEA before and after 530℃ annealing process. **a**) XRD patterns of the CoNiV samples after various annealing durations at 530℃. **b,c**) The IPF maps of RC and AL, respectively.


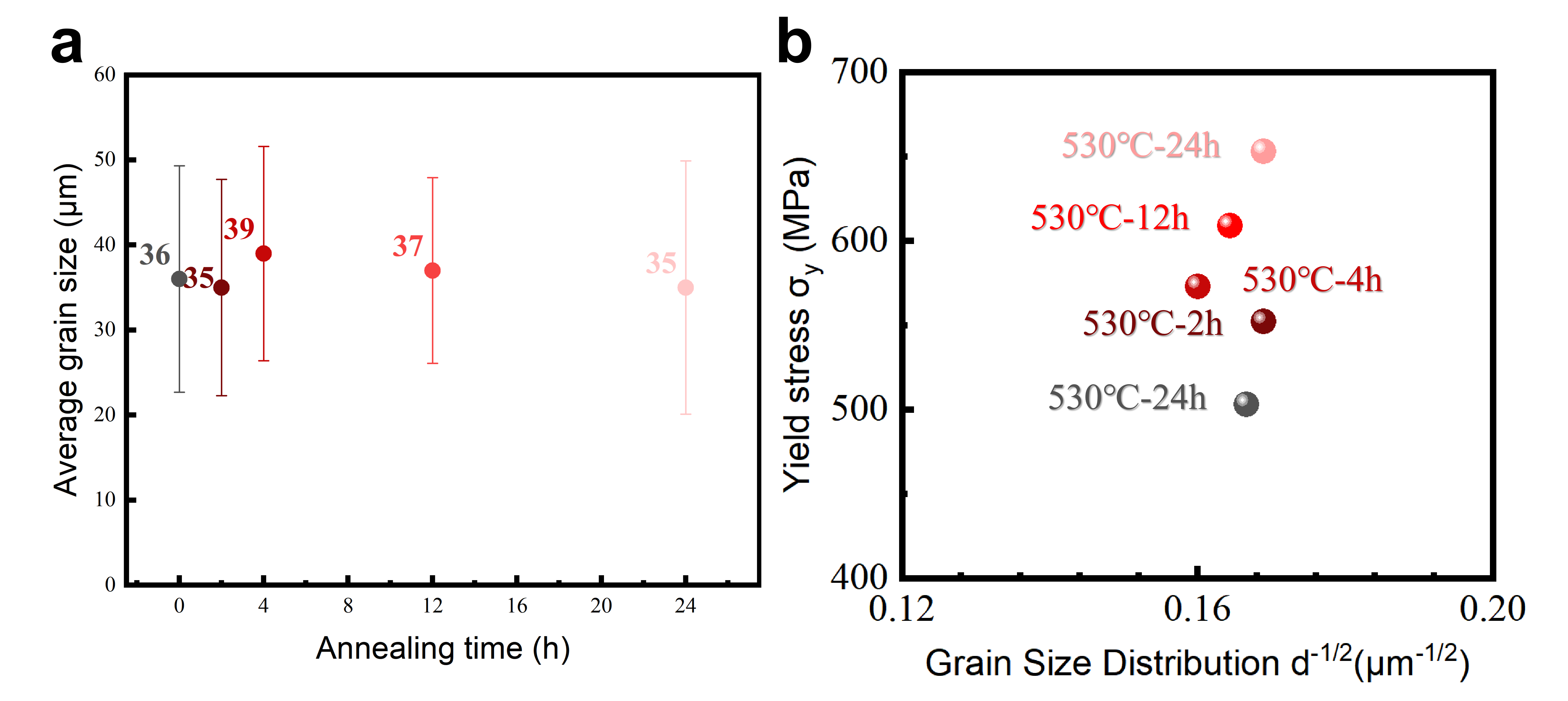


**Figure S2. a**) The average grain size of CoNiV samples before and after 530℃ annealing. **b**) Hall-Petch relationship of the CoNiV MEAs.


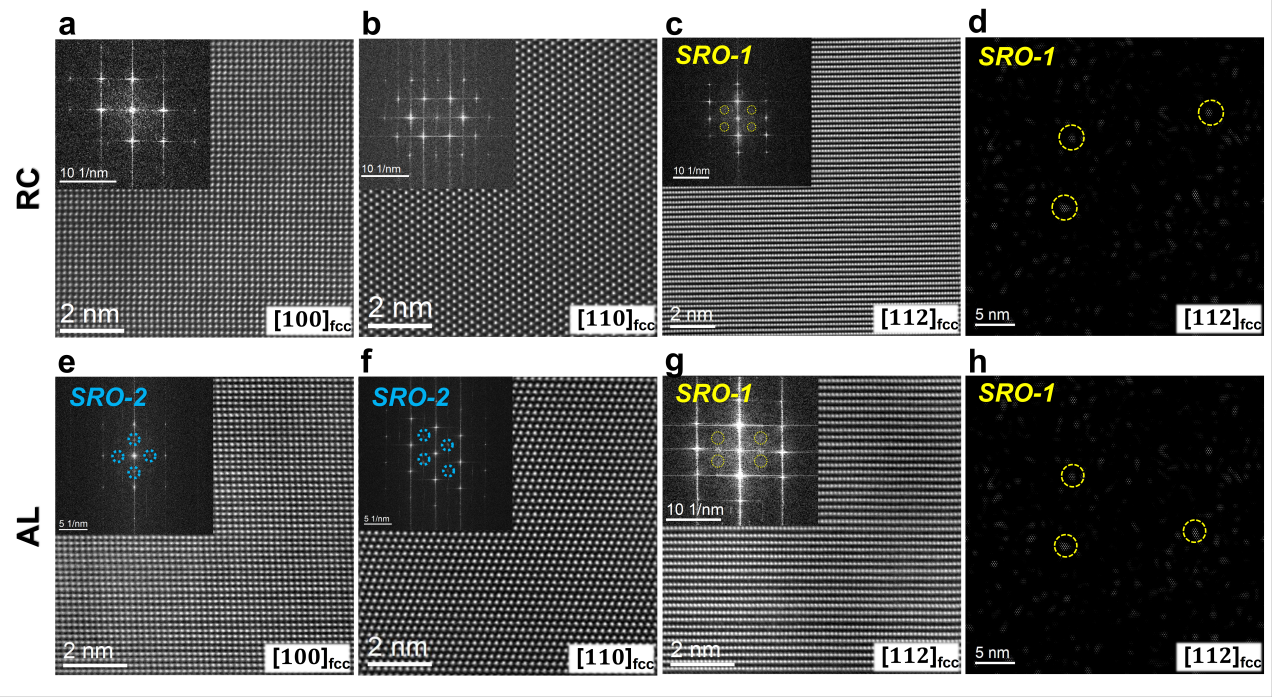


**Figure S3.** Atomic structure of RC and AL. **a-c**) The STEM-HAADF images of RC along the [$100$], [$110$] and [$112$] zone axes, respectively, with the corresponding FFT patterns as the inserts, showing additional diffuse reflections of SRO-1 (circled in yellow). **d**) IFFT image revealing the illumination of SRO-1 regions in RC (circled in yellow). **e-g**) The STEM-HAADF images of AL along the $[100]$, $[110]$ and $[112]$ zone axes, respectively, with the corresponding FFT patterns as the inserts, showing additional diffuse reflections of SRO-1 (circled in yellow) and SRO-2 (circled in blue), respectively. **h**) IFFT image revealing the illumination of SRO-1 regions in AL (circled in yellow).


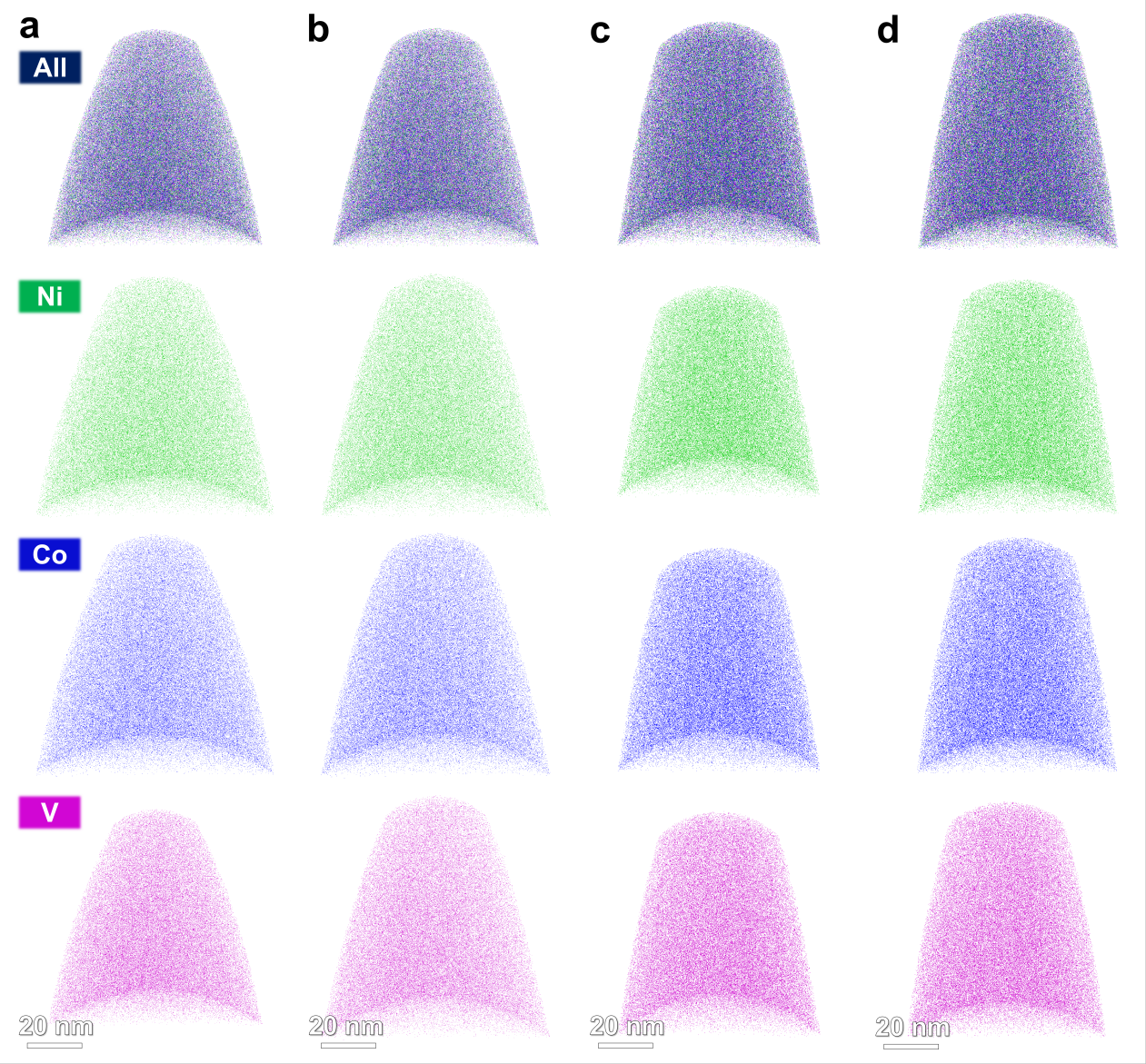


**Figure S4. a-d**) 3D-reconstruction profiles of CoNiV annealed at 530℃ for 0, 2, 12, and 24 hours, respectively. Green, blue and purple colors correspond to Ni, Co, and V atoms, respectively.


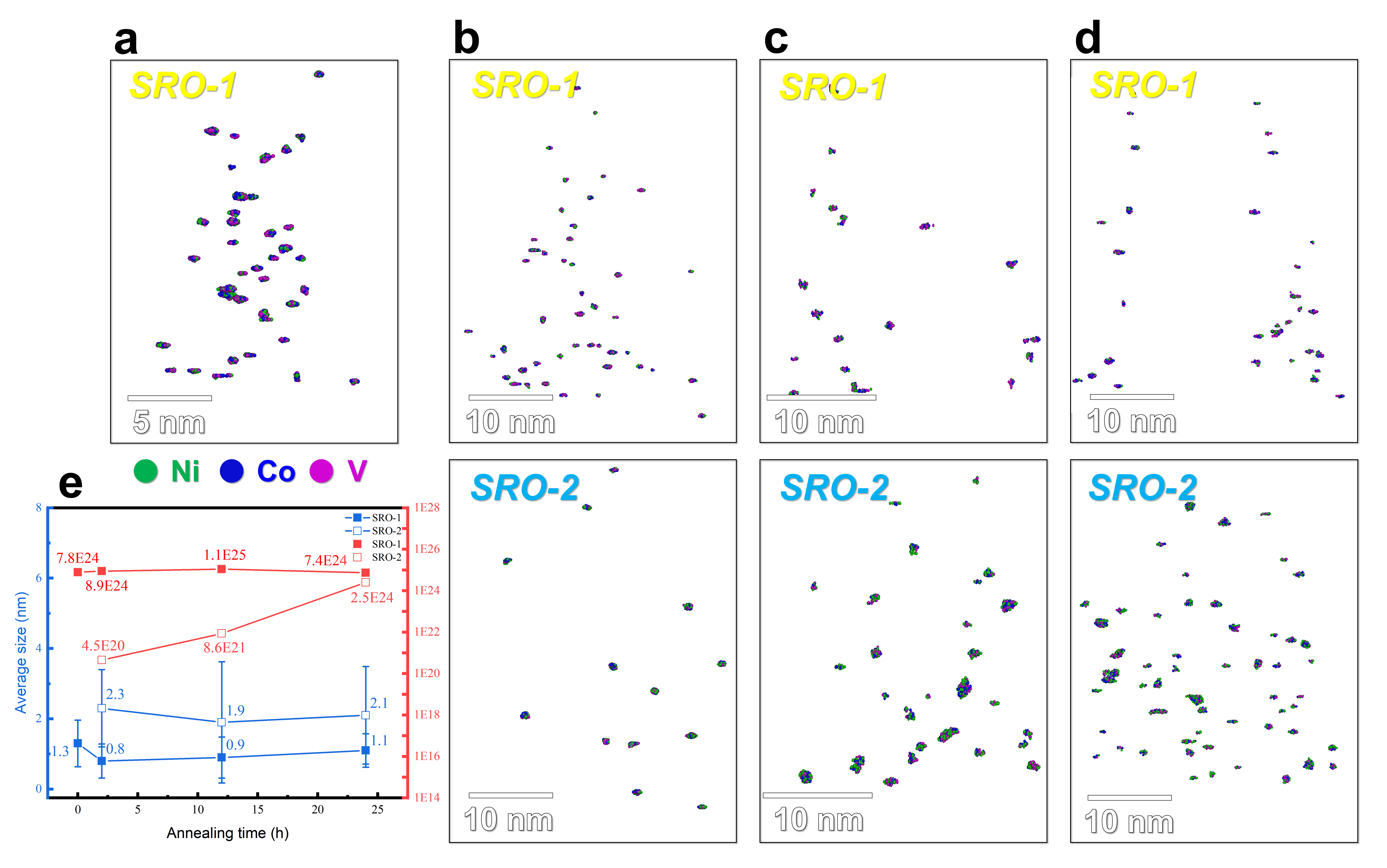


**Figure S5. a-d**) 3D-reconstruction profiles of SROs annealed at 530℃ for 0, 2, 12, and 24 hours, respectively. Green, blue and purple colors correspond to Ni, Co, and V atoms, respectively. **e**) Average size and number density of SRO-1 and SRO-2 before and after 530℃ annealing.


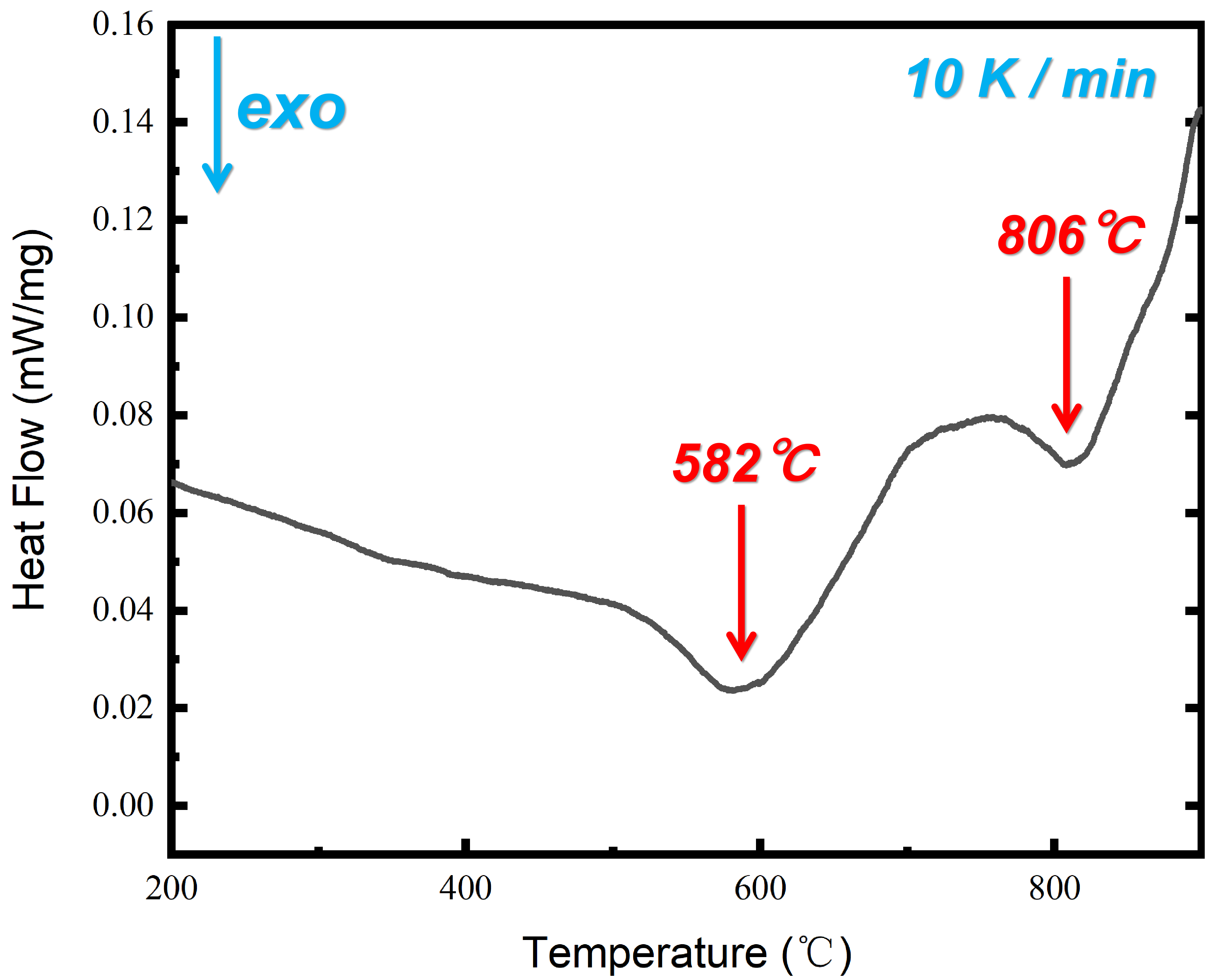


**Figure S6.** DSC curve of CoNiV heating from 200℃ to 900℃, exothermic peaks arrowed in red appear at 582℃ and 806℃, indicating phase transition.


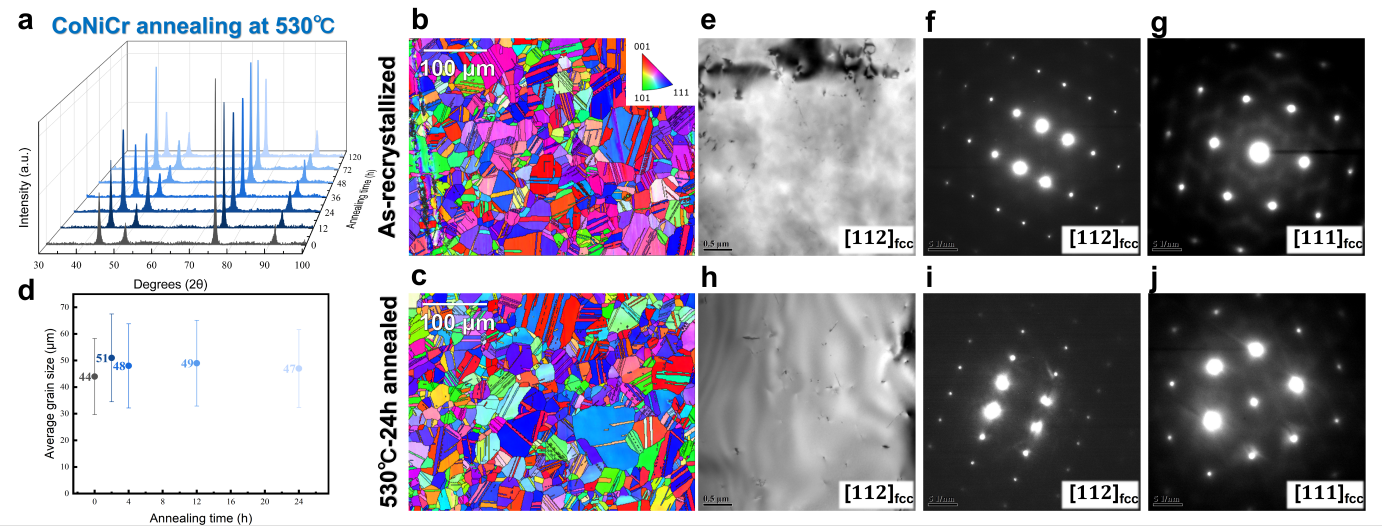


**Figure S7.** Structure and phase constitution of the CoNiCr MEA before and after 530℃ annealing process. **a**) XRD patterns of the CoNiCr samples after various annealing durations at 530℃. **b,c**) The IPF maps of RC and AL, respectively. **d**) The average grain size of CoNiCr samples before and after 530℃ annealing. **e,h**) Bright-field TEM images of the as-recrystallized and 530℃-24 hours-annealed samples from the [$112$] z.a., respectively. **f,g**) and **i,j**) The SAED patterns of the as-recrystallized and the 530℃-24 hours-annealed CoNiCr MEA along the $[112]$, $[111]$ z.a., respectively.


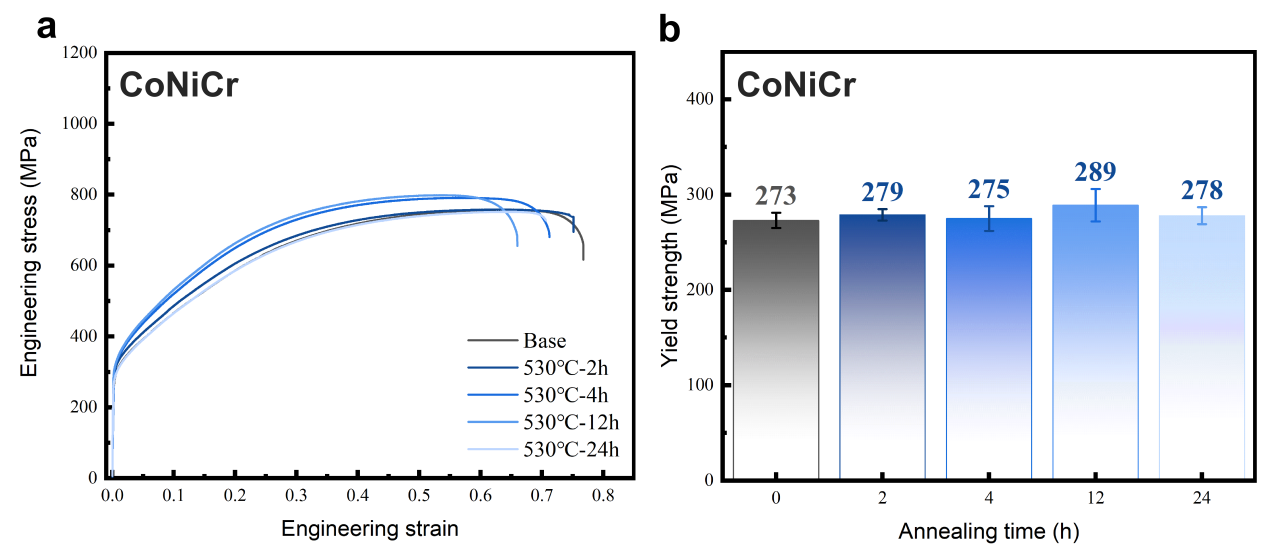


**Figure S8.** Tensile properties of the CoNiCr MEA before and after 530℃ annealing process. **a)** Engineering stress-strain curves of the recrystallized and the annealed CoNiCr MEA. **b)** The yield strength values ($\sigma_{y}$) of CoNiCr samples as the annealing time prolonged.


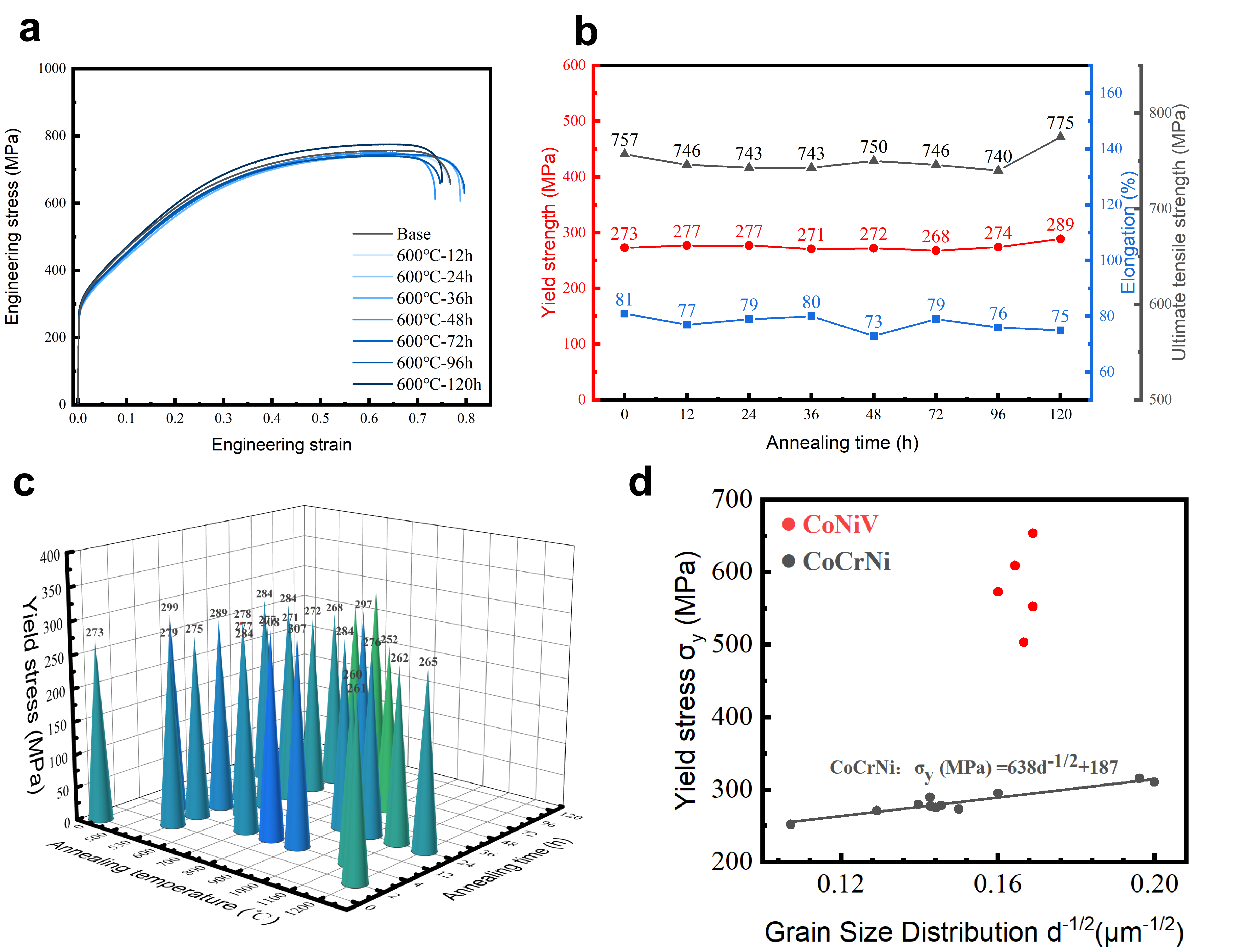


**Figure S9.** Tensile properties of the CoNiCr MEA before and after annealing. **a**) Engineering stress-strain curves of the CoNiCr MEA in recrystallized state and after 600℃ annealing. **b**) Evolution of yield strength ($\sigma_{y}$), ultimate tensile strength ($\sigma_{\mathrm{UTS}}$), and elongation ($\varepsilon$) with prolonged annealing time at 600℃. **c**) Three-dimensional bar chart comparing yield strength ($\sigma_{y}$) of CoNiCr samples under different annealing conditions. **d**) Hall-Petch relationship of CoNiCr MEAs versus CoNiV MEAs.


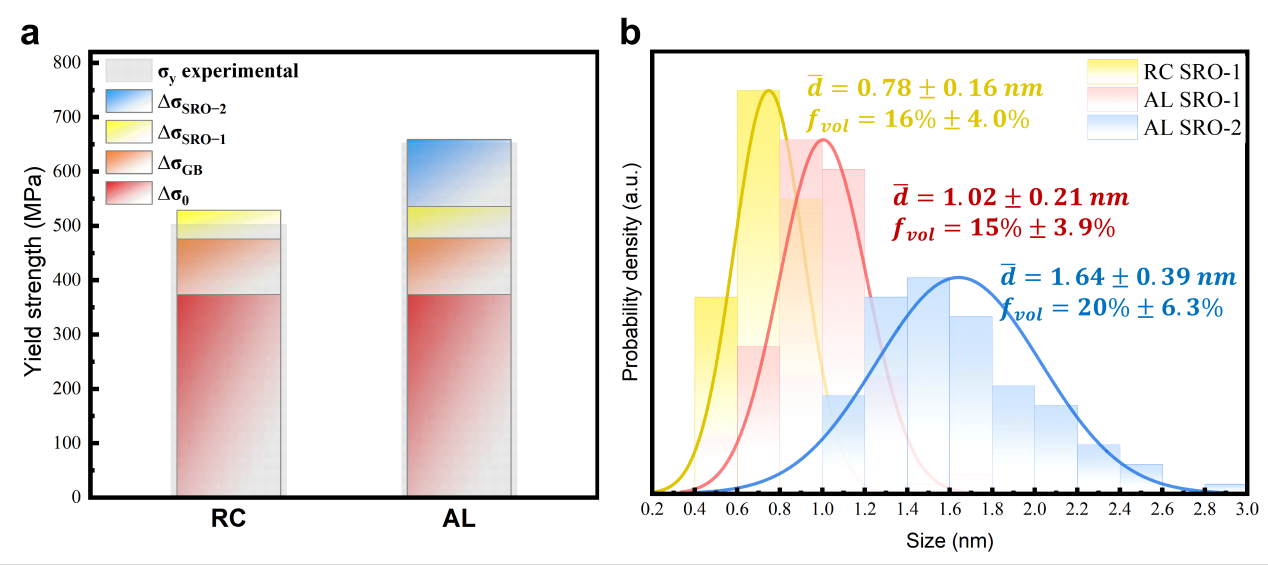


**Figure S10.** Strengthening contributions of CoNiV. **a**) Strengthening contributions from different strengthening mechanisms in RC and AL. **b**) Quantitative statistics of multi-scale SROs in RC and AL.


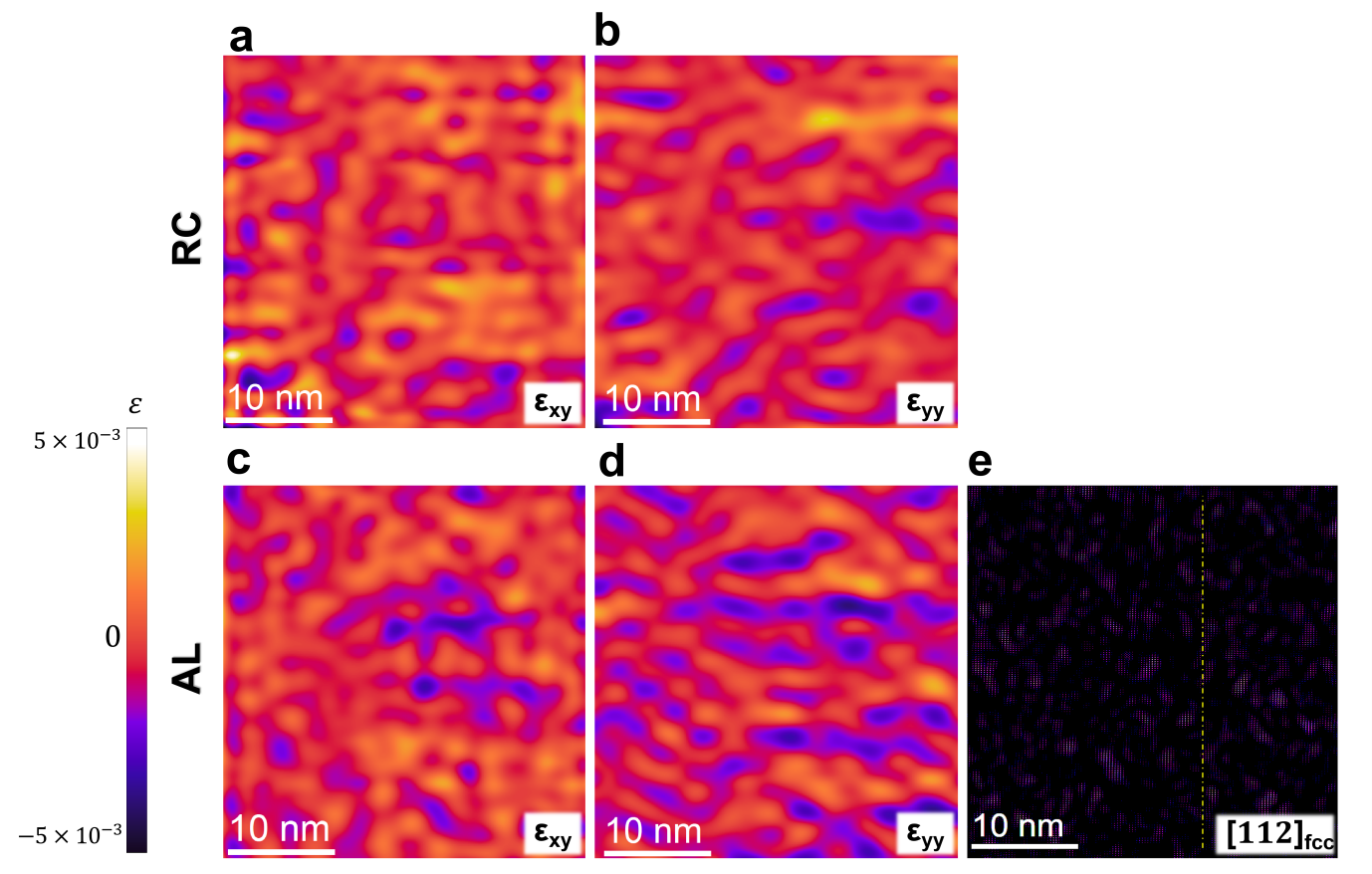


**Figure S11.** Strain field analysis of RC and AL before tensile deformation. **a,b**) Atomic-strain mapping of ε_xy_ and ε_yy_ for RC along the [$112$] z.a., respectively. **c,d**) Atomic-strain mapping of ε_xy_ and ε_yy_ for AL along the [$112$] z.a., respectively. **e**) The corresponding IFFT image for AL, and the relative position of line scan region corresponding to **Figure 4e**.

**Supporting Note**

**Size-Distribution Discrepancies of SROs in STEM-HAADF and APT Analyses**

The differences in measured size and distribution of SROs between STEM-HAADF and APT stem from their distinct detection principles and resolution capabilities. STEM-HAADF, a Z-contrast imaging technique, detects SROs via local atomic number contrast variations but may average over small clusters due to its ~0.1-0.2 nm resolution limit. APT, offering near-atomic 0.3-0.5 nm resolution, provides direct 3D atomic-scale mapping but may underestimate cluster sizes due to trajectory aberrations. Additionally, STEM-HAADF samples in-plane ordering in thin foil specimens, whilst APT captures the full 3D distribution, potentially leading to different statistical representations of the same microstructure.

Key findings in this study from both techniques include:

- **SRO-1**: Demonstrates stable volume fraction (~16%→15%, see **Figure S3d,h**) with slight size growth (0.78→1.02 nm via STEM-HAADF, see **Figure S3d,h**; 1.3→1.1 nm via APT, see **Figure S5e**).
- **SRO-2**: Shows low-temperature activation, forming 1.64 nm clusters (20% volume fraction) via STEM-HAADF (see **Figure 2j**) and larger 2.1 nm clusters (2.5E24 number density) via APT (see **Figure S5e**).

These variations are within the measurement error ranges of the complementary techniques. The combined use of STEM-HAADF and APT provides robust, cross-validated evidence for understanding SRO evolution.

**References**

[S1] B. Gludovatz, A. Hohenwarter, D. Catoor, E. H. Chang, E. P. George, R. O. Ritchie, Science **2014**, 345, 6201.

[S2] Z. Wu, H. Bei, G. M. Pharr, E. P. George, Acta Mater. **2014**, 81, 428.

[S3] Z Li, S. Ma, S. Zhao, W. Zhang, F. Peng, Q. Li, T. Yang, C.-Y. Wu, D. Wei, Y.-C. Chou, P. K. Liaw, Y. Gao, Z. Wu, Mater. Today **2023**, 63, 10.

[S4] T. J. Jang, W. S. Choi, D. W. Kim, G. Choi, H. Jun, A. Ferrari, F. Körmann, P.-P. Choi, S. S. Sohn, Nat. Commun. **2021**, 12, 1.

[S5] J. Y. He, W. H. Liu, H. Wang, Y. Wu, X. J. Liu, T. G. Nieh, Z. P. Lu, Acta Mater. 2014, 62, 105.

[S6] Z. Wang, I. Baker, W. Guo, J. D. Poplawsky, Acta Mater. 2017, 126, 346.

[S7] A. J. Zaddach, R. O. Scattergood, C. C. Koch, Mater. Sci. Eng. A 2015, 636, 373.

[S8] M. Yao, K. G. Pradeep, C. C. Tasan, D. Raabe, Scr. Mater. 2014, 72, 5.

[S9] L. B. Chen, R. Wei, K. Tang, J. Zhang, F. Jiang, L. He, J. Sun, Mater. Sci. Eng. A 2018, 716, 150.

[S10] W. H. Liu, Z. P. Lu, J. Y. He, J. H. Luan, Z. J. Wang, B. Liu, C. T. Liu, Acta Mater. 2016, 116, 332.

[S11] K. Ming, X. Bi, J. Wang, Scr. Mater. 2017, 137, 88.

[S12] J. W. Bae, J. M. Park, J. Moon, W. M. Choi, B.-J. Lee, H. S. Kim, J. Alloys Compd. 2019, 781, 75.

[S13] S. Shukla, D. Choudhuri, T. Wang, K. Liu, R. Wheeler, S. Williams, B. Gwalani, R. S. Mishra, Mater. Res. Lett. 2018, 6, 676.

[S14] S. Niu, H. Kou, T. Guo, Y. Zhang, J. Wang, J. Li, Mater. Sci. Eng., A 2016, 671, 82.

[S15] J. C. Rao, H. Y. Diao, V. Ocelík, D. Vainchtein, C. Zhang, C. Kuo, Z. Tang, W. Guo, J. D. Poplawsky, Y. Zhou, P. K. Liaw, J. Th. M. De Hosson, Acta Mater. 2017, 131, 206.

[S16] I. S. Wani, T. Bhattacharjee, S. Sheikh, P. P. Bhattacharjee, S. Guo, N. Tsuji, Mater. Sci. Eng., A 2016, 675, 99.

[S17] Q. Wang, Y. Ma, B. Jiang, X. Li, Y. Shi, C. Dong, P. K. Liaw, Scr. Mater. 2016, 120, 85.

[S18] Z. G. Wang, W. Zhou, L. M. Fu, J. F. Wang, R. C. Luo, X. C. Han, B. Chen, X. D. Wang, Mater. Sci. Eng., A 2017, 696, 503.

[S19] E. P. George, W. A. Curtin, C. C. Tasan, Acta Mater. 2020, 188, 435.

[S20] H. Huang, Y. Wu, J. He, H. Wang, X. Liu, K. An, W. Wu, Z. Lu, Adv. Mater. 2017, 29, 1701678.

[S21] O. N. Senkov, S. L. Semiatin, J. Alloys Compd. 2015, 649, 1110.

[S22] S. Sheikh, S. Shafeie, Q. Hu, J. Ahlström, C. Persson, J. Veselý, J. Zýka, U. Klement, S. Guo, J. Appl. Phys. 2016, 120, 164905.

[S23] Y. Wu, Mater. Sci. Eng. A 2018, 724, 249.

[S24] S. S. Sohn, A. Kwiatkowski da Silva, Y. Ikeda, F. Körmann, W. Lu, W. S. Choi, B. Gault, D. Ponge, J. Neugebauer, D. Raabe, Adv. Mater. 2019, 31, 1807142.

[S25] T.-T. Shun, Y.-C. Du, J. Alloys Compd. 2009, 479, 157.

[S26] S. Shukla, D. Choudhuri, T. Wang, K. Liu, R. Wheeler, S. Williams, B. Gwalani, R. S. Mishra, Mater. Res. Lett. 2018, 6, 676.

[S27] D. Li, C. Li, T. Feng, Y. Zhang, G. Sha, J. J. Lewandowski, P. K. Liaw, Y. Zhang, Acta Mater. 2017, 123, 285.

[S28] Y. Lu, X. Gao, L. Jiang, Z. Chen, T. Wang, J. Jie, H. Kang, Y. Zhang, S. Guo, H. Ruan, Y. Zhao, Z. Cao, T. Li, Acta Mater. 2017, 124, 143.

[S29] C.-W. Tsai, M.-H. Tsai, K.-Y. Tsai, S.-Y. Chang, J.-W. Yeh, A.-C. Yeh, Mater. Sci. Technol. 2015, 31, 1178.

[S30] J. M. Park, J. Moon, J. W. Bae, J. Jung, S. Lee, H. S. Kim, Mater. Sci. Eng. A 2018, 728, 251.

[S31] K. Sohlberg, T. J. Pennycook, W. Zhou, S. J. Pennycook, Phys. Chem. Chem. Phys. 2015, 17, 3982.

[S32] B. Gault, A. Chiaramonti, O. Cojocaru-Mirédin, P. Stender, R. Dubosq, C. Freysoldt, S. K. Makineni, T. Li, M. Moody, J. M. Cairney, Nat. Rev. Methods Primers 2021, 1, 1.

[S33] Y.-J. Kim, R. Tao, R. F. Klie, D. N. Seidman, ACS Nano 2012, 7, 732.
